# Squaramides enhance NLRP3 inflammasome activation by lowering intracellular potassium

**DOI:** 10.1038/s41420-023-01756-9

**Published:** 2023-12-22

**Authors:** Paula I. Seoane, James A. Beswick, Andrew G. Leach, Tessa Swanton, Lucy V. Morris, Kevin Couper, Martin Lowe, Sally Freeman, David Brough

**Affiliations:** 1 Division of Neuroscience, School of Biological Sciences, Faculty of Biology, Medicine and Health, Manchester Academic Health Science Centre, University of Manchester, Manchester, UK; 2 Geoffrey Jefferson Brain Research Centre, The Manchester Academic Health Science Centre, Northern Care Alliance NHS Group, University of Manchester, Manchester, UK; 3 The Lydia Becker Institute of Immunology and Inflammation, University of Manchester, Manchester, UK; 4 Division of Pharmacy and Optometry, School of Health Sciences, Faculty of Biology, Medicine and Health, Manchester Academic Health Science Centre, University of Manchester, Manchester, UK; 5 Biodiscovery Institute, University Park, University of Nottingham, Nottingham, UK; 6 The Francis Crick Institute, London, UK; 7 Division of Infection, Immunity & Respiratory Medicine, School of Biological Sciences, Faculty of Biology, Medicine and Health, University of Manchester, Manchester, UK; 8 Division of Molecular and Cellular Function, School of Biological Sciences, Faculty of Biology, Medicine, and Health, Manchester Academic Health Science Centre, University of Manchester, Manchester, UK

**Keywords:** Phagocytes, Inflammasome, Small molecules

## Abstract

The NLRP3 inflammasome is a component of the inflammatory response to infection and injury, orchestrating the maturation and release of the pro-inflammatory cytokines interleukin-1β (IL-1β), IL-18, and triggering pyroptotic cell death. Appropriate levels of NLRP3 activation are needed to avoid excessive tissue damage while ensuring host protection. Here we report a role for symmetrical diarylsquaramides as selective K^+^ efflux-dependent NLRP3 inflammasome enhancers. Treatment of macrophages with squaramides potentiated IL-1β secretion and ASC speck formation in response to K^+^ efflux-dependent NLRP3 inflammasome activators without affecting priming, endosome cargo trafficking, or activation of other inflammasomes. The squaramides lowered intracellular K^+^ concentration which enabled cells to respond to a below-threshold dose of the inflammasome activator nigericin. Taken together these data further highlight the role of ion flux in inflammasome activation and squaramides as an interesting platform for therapeutic development in conditions where enhanced NLRP3 activity could be beneficial.

## Introduction

Inflammasomes are multimeric protein complexes which assemble in immune cells upon disruption of cellular homeostasis (during infection or injury), and drive the processing and release of pro-inflammatory cytokines IL-1β and IL-18. Inflammasome activation also results in pyroptosis, a form of inflammatory cell death [1]. The best studied inflammasome is formed by the cytosolic pattern recognition receptor (PRR) NLR family pyrin domain containing 3 (NLRP3), the adaptor protein apoptosis-associated speck-like protein containing a caspase-recruitment domain (ASC) and the enzyme pro-caspase-1. Upon activation, NLRP3 recruits the adaptor protein ASC which oligomerises into a large speck and in turn recruits pro-caspase-1 which becomes activated and directly causes the maturation of pro-IL-1β and pro-IL-18 into their active forms. Caspase-1 also cleaves gasdermin D, which forms pores in the plasma membrane and facilitates release of IL-1β and IL-18, and leads to pyroptosis [2].

The NLRP3 inflammasome is activated by diverse stimuli ranging from pathogen-associated or damage-associated molecular patterns (PAMPs or DAMPs, respectively) such as the bacterial toxin nigericin or the presence of extracellular ATP [3], to the disruption of lysosomal integrity by particulate material [4]. Recently, our group and others have reported NLRP3 acting as a sensor of organelle dysfunction [5, 6]. Many known activators of the NLRP3 inflammasome induce potassium (K^+^) efflux from the cytoplasm [7]. K^+^ efflux is coupled with movement of chloride (Cl^−^), which by itself is sufficient for ASC oligomerisation in macrophages [8].

Over-activation of the inflammasome is linked to multiple diseases, including Alzheimer’s disease, atherosclerosis and autoinflammatory conditions such as Cryopyrin-associated periodic syndrome (CAPS) [9]. On the other hand, absence of inflammasome components renders mice more susceptible to infection [10, 11], highlighting the importance of a well-regulated inflammasome system. Thus, molecules capable of modulating inflammasome activation are of interest. Our group previously reported a series of molecules containing a urea linker capable of inhibiting K^+^ efflux-dependent NLRP3 inflammasome activation [12]. Here we extend this series to investigate the effects of molecules with a squaramide linker on inflammasome activation.

Squaramides are small organic compounds featuring a four carbon “square” ring. They are easy to synthesize in good yield, which makes them a desirable platform for the development of therapeutics and experimental reagents. When compared to analogous thioureas or ureas, squaramides have shown improved anion transport across lipid bilayers, with compounds having the electron-withdrawing trifluoromethyl groups being the most active [13]. Data from computational and experimental approaches suggests squaramides transport anions across lipid bilayers in a mobile carrier fashion rather than creating a channel, with Cl^−^/nitrate or Cl^−^/bicarbonate exchange [13, 14]. In a cellular model, the small symmetrical bis(4-(trifluoromethyl)phenyl) squaramide disrupts autophagy and induces apoptosis by altering cellular Cl^−^ concentrations [15]. To-date the impact of squaramides on inflammasome activation has not been explored. Given their anion transport capability we hypothesised that squaramide compounds could affect inflammasome activation by altering ion movement.

Here we describe the effects of small electron-donating symmetrical squaramides on the activation of the NLRP3 inflammasome. The selected squaramides enhanced IL-1β secretion induced by K^+^ efflux-dependent stimuli. The squaramides did not impact cell viability on their own and acted by lowering intracellular K^+^ levels, thus sensitising cells to the NLRP3 inflammasome activating stimuli.

## Results

### Squaramides enhance NLRP3 inflammasome activation

**Fig. 1:**
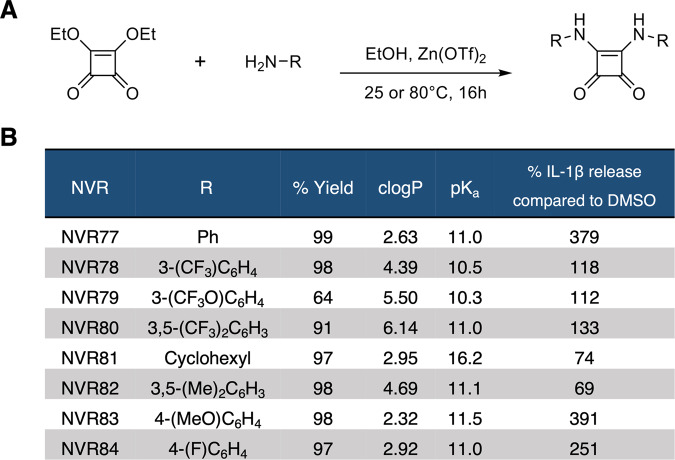
Screen of squaramide NVR compounds. **A** General schematic for the synthesis of squaramide NVR compounds. **B** Summary data for synthesis yield, calculated clogP and pKa of the synthesised compounds and their effect on nigericin-induced IL-1β secretion in BMDMs shown as %IL-1β released compared to DMSO control. See also Supplementary information for detailed synthesis data and Fig. S1 for IL-1β secretion and LDH release raw data.

Our group previously reported the inhibitory effect of urea-based fenamate analogues (which we designated NVR compounds) on the NLRP3 inflammasome [12]. Additional analogues containing a squaramide linker were developed as part of the NVR series which remained unpublished. The synthesis for the squaramide compound library is shown in general here (Fig. 1A) and described in detail here (Supplementary information). The effect of squaramides on inflammasome activation was screened using primary mouse bone marrow-derived macrophages (BMDMs). LPS-primed BMDMs were treated with 10 μM of the squaramide compounds labelled NVR77-84 or DMSO vehicle control for 15 minutes and subsequently activated with the NLRP3 inflammasome activator nigericin for 90 minutes with IL-1β release measured (Fig. 1B). In the absence of nigericin, none of the squaramides tested caused IL-1β release or had a significant impact on cell viability as assessed by LDH release (Fig. S1). In the presence of nigericin however, some of the squaramides significantly enhanced nigericin-induced IL-1β release (Fig. 1B and S1). Notably, squaramides NVR77 and NVR83 were identified as strong enhancers of nigericin-induced IL-1β release (Fig. 1B and S1) and were carried through for further investigation.

**Fig. 2:**
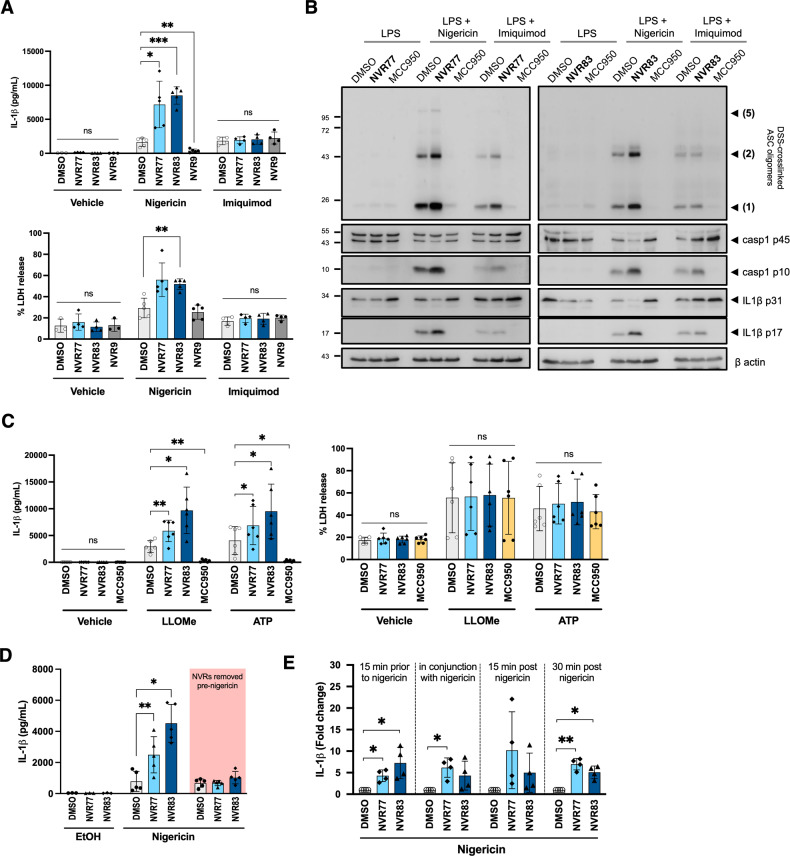
Squaramides enhance NLRP3 activation. **A** LPS-primed (1 μg/mL; 4 h) BMDMs were pre-treated with 10 μM NVR compound or vehicle control (DMSO 0.5% v/v) for 15 minutes before addition of 10 μM nigericin or 75 μM imiquimod. After 90 minutes, culture supernatants were recovered and probed for IL-1β content and LDH release. Data correspond to mean +/− SD of 5 biological repeats. See also Fig. S2 and S3 for dose-response data of NVR77 and NVR83 in conjunction with nigericin or imiquimod. **B** Cells were treated as in A. After 90 minutes, cells were lysed in well with 1% v/v triton X-100. The triton-insoluble fraction was subsequently DSS-crosslinked and probed for ASC oligomer formation. The triton-soluble fraction was probed for caspase-1 cleavage, IL-1β cleavage and βactin content. Western blots shown are representative of at least 3 biological repeats. **C** LPS-primed (1 μg/mL; 4 h) BMDMs were pre-treated with 10 μM NVR compound, 10 μM MCC950 or vehicle control (DMSO 0.5% v/v) for 15 minutes before addition of 1 mM LLOMe or 5 mM ATP. After 90 min, cell supernatants were recovered and probed for IL-1β content and LDH release. Data correspond to mean +/− SD of 5 biological repeats. **D** LPS-primed (1 μg/mL; 4 h) BMDMs were pre-treated with 10 μM NVR compound or vehicle control (DMSO 0.5% v/v) for 15 minutes. The NVRs were then washed off the cells (pink box) or left present during stimulation with 10 μM nigericin. After 90 minutes, cell supernatants were recovered and probed for IL-1β content. Data correspond to mean +/− SD of 5 biological repeats. **E** LPS-primed (1 μg/mL; 4 h) BMDMs were stimulated with 10 μM nigericin for 90 min. Cells were also treated with 10 μM NVRs or DMSO control (0.5% v/v) added 15 min prior nigericin, in conjunction with nigericin or 15 or 30 min post-nigericin. Cell supernatants were recovered and probed for IL-1β content. Data correspond to mean +/− SD of IL-1β secretion normalised to Nigericin + DMSO control from 4 biological repeats. Statistically significant differences were assessed by two-way ANOVA and Dunnett’s post-test. Differences in normalised data were assessed by multiple t-tests and corrected for multiple comparisons using Holm-Sidak method. *p < 0.05, **p < 0.01, ***p < 0.001.

The squaramides NVR77 and NVR83 were tested alongside the non-squaramide compound NVR9, one of our previously reported NLRP3 inhibitors [12], on LPS-primed BMDMs activated with 10 μM nigericin or 75 μM of the K^+^ efflux-independent stimulus imiquimod. The results of the initial screen were reproduced and showed that both NVR77 and NVR83 squaramides strongly potentiated nigericin-induced IL-1β secretion and LDH release (Fig. 2A and S2) unlike the urea compound NVR9, highlighting the peculiar property of the squaramide motif (Fig. 2A). Imiquimod activates the NLRP3 inflammasome independently of K^+^ efflux [16], whereas most other well studied NLRP3 inflammasome activators, including nigericin, depend upon K^+^ efflux [7]. Our previously reported NVR inhibitor series, which we proposed act by inhibiting Cl^−^ transport, selectively inhibited NLRP3 activation in response to K^+^ efflux dependent agonists [12]. Neither NVR77 nor NVR83 potentiated imiquimod-induced IL-1β secretion or LDH release (Fig. 2A and S3), suggesting that the effect was selective for K^+^ efflux-dependent activators. Consistent with the effect on IL-1β release, both squaramides potentiated nigericin-induced ASC oligomerisation, cleavage of caspase-1 and of IL-1β (Fig. 2B).

Next we assessed the impact of the squaramides NVR77 and NVR83 on the activation of the NLRP3 inflammasome induced by other K^+^ efflux-dependent activators, namely the lysosomal disrupting agent L-leucyl-L-leucine methyl ester (LLOMe) [4] and extracellular ATP [3] (Fig. 2C). As described above, LPS-primed BMDMs were treated with 10 μM squaramide NVR compounds or vehicle for 15 min, and subsequently activated with 1 mM LLOMe or 5 mM ATP for a further 90 min. The NLRP3 inhibitor MCC950 [17] was used as a control. NVR77 and NVR83 enhanced IL-1β secretion in response to LLOMe and ATP and this was inhibited by MCC950 (Fig. 2C). The squaramides did not potentiate the LDH release induced by these activators, which was not NLRP3-dependent as it was not inhibited by MCC950 (Fig. 2C).

The potentiation of NLRP3 inflammasome activation by the squaramides NVR77 and NVR83 was reversible as removal of the compounds just prior to nigericin treatment abolished the potentiation (Fig. 2D). Furthermore, addition of the squaramides after nigericin, instead of 15 min prior, also resulted in a potentiation effect (Fig. 2E).

### NVR77 and NVR83 enhance nigericin-induced ASC-speck formation

**Fig. 3:**
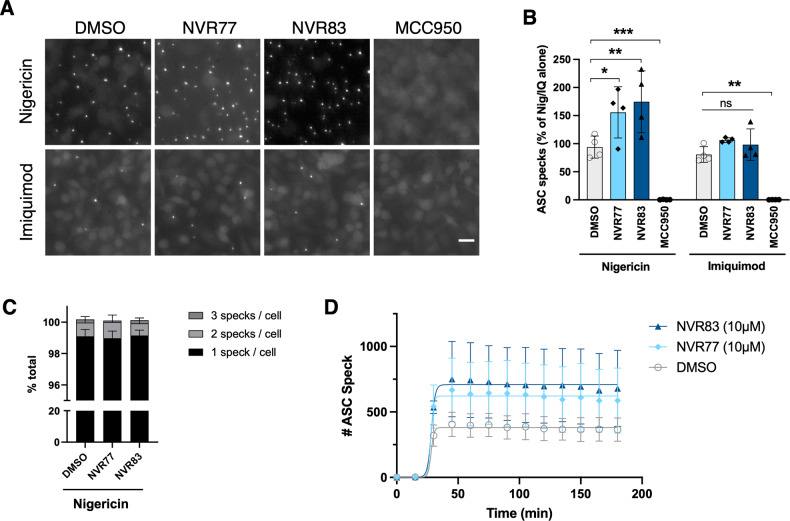
Squaramides potentiate ASC speck formation induced by nigericin. LPS-primed (1 μg/mL; 4 h) ASC-citrine BMDMs were treated with 10 μM NVR compound, vehicle control (DMSO 0.5% v/v) or 10 μM MCC950 in conjunction with 10 μM nigericin or 75 μM imiquimod in OptiMEM media. Immediately after addition of stimuli cells were placed on a live imaging fluorescence microscope. Images were taken every 15 min over a period of 3 hours. **A** Representative images for the stated conditions at 90 minutes post inflammasome activation. Scale bar is 20 μm. **B** Quantification of ASC specks formed at 90 minutes post inflammasome activation. Data shown as mean +/− SD of 4 biological repeats normalised to nigericin or imiquimod alone condition. Differences were assessed by two-way ANOVA and Dunnett’s post-test. *p < 0.05, **p < 0.01, ***p < 0.001. **C** Quantification of ASC specks per cell at 90 min post addition of 10 μM nigericin in conjunction with 10 μM NVR compound or vehicle control. Data shown as mean +/− SD of 4 biological repeats. **D** Number of ASC specks formed in response to 10 μM nigericin in the presence of 10 μM NVR compound or vehicle control over time. Data shown as mean +/− SD of 4 biological repeats and fitted to a sigmoid curve.

The effect of squaramides NVR77 and NVR83 on ASC speck formation was next assessed in primary BMDMs isolated from ASC-citrine expressing mice [18]. LPS-primed ASC-citrine BMDMs were treated with 10 μM squaramide NVR77 or NVR83 or vehicle control in conjunction with 10 μM nigericin or 75 μM imiquimod. Formation of ASC specks was assessed using live-cell fluorescence imaging as described previously [19] (Fig. 3). Consistent with the ASC oligomerisation data (Fig. 2B), the number of ASC specks formed in response to nigericin, but not imiquimod, significantly increased in the presence of NVR77 or NVR83 (Fig. 3A, B). The increase in number of ASC specks was not explained by a higher number of specks per cell, but rather an overall rise in the number of cells responding to nigericin by forming an ASC speck, with each cell predominantly containing only one ASC speck (Fig. 3C). The number of ASC specks formed over time for cells treated with nigericin in the presence of the squaramide compounds or vehicle was plotted and fitted to a sigmoid curve (Fig. 3D). Notably, the presence of the squaramides resulted in a higher number of ASC specks overall but did not affect the rate of ASC speck formation (Fig. 3D).

### NVR77 and NVR83 selectively potentiate the NLRP3 inflammasome

**Fig. 4:**
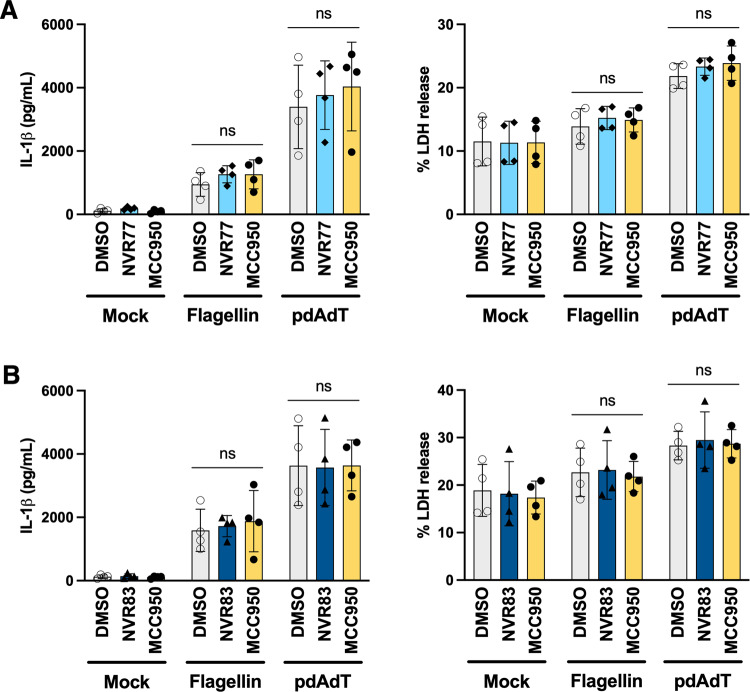
Squaramides do not affect NLRC4 or AIM2 inflammasome activation. LPS-primed (1 μg/mL; 4 h) BMDMs were pre-treated with 10 μM NVR compound, vehicle control (DMSO 0.5% v/v) or 10 μM MCC950 for 15 min before being transfected with either 1 μg/mL flagellin or 1 μg/mL pdAdT using lipofectamine 3000 and OpitMEM media. After 3 h, supernatants were recovered and probed for IL-1β content by ELISA (left) and LDH release (right). Data correspond to mean +/− SD of 4 biological repeats. Differences were assessed by Two-way ANOVA followed by Dunnett’s post-test. **A** IL-1β release (left) and LDH release (right) induced by NLRC4 or AIM2 inflammasome activation in the presence or absence of 10 μM NVR77. **B** IL-1β release (left) and LDH release (right) induced by NLRC4 or AIM2 inflammasome activation in the presence or absence of 10 μM NVR83.

NVR77 and NVR83 were also tested against the activation of inflammasome forming cytosolic PRRs NLRC4 and AIM2. Activation of NLRC4 and AIM2 inflammasomes is dependent on the recognition of specific PAMPs; NLRC4 is activated in the presence of cytosolic flagellin from *Salmonella typhimurium* [20, 21] and AIM2 is activated in the presence of cytosolic double stranded DNA [22, 23]. BMDMs were primed with LPS and treated with 10 μM NVR77 or NVR83 or vehicle for 15 min. The cells were then transfected with either flagellin to activate NLRC4 or with DNA mimetic polydA:dT to activate AIM2 using lipofectamine, in the presence of the squaramide NVR compounds or vehicle, for a further three hours. Neither NLRC4 nor AIM2 inflammasome-dependent IL-1β or LDH release were influenced by NVR77 or NVR83 (Fig. 4A, B).

### Squaramides do not affect priming nor endosome cargo trafficking

**Fig. 5:**
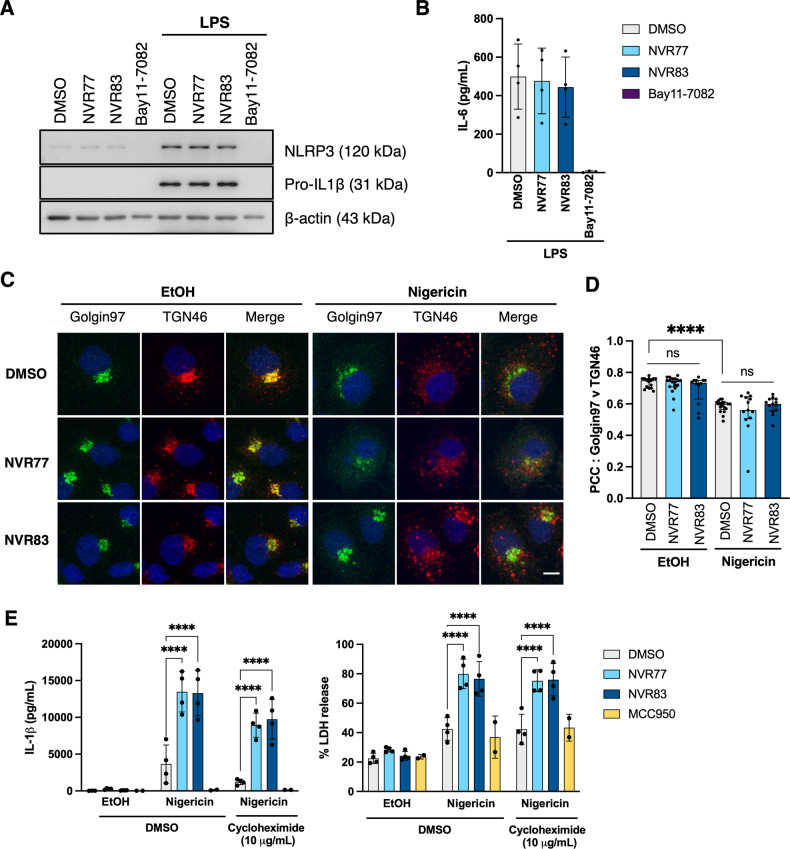
The effects of squaramides on other NLRP3 activation mechanisms. **A** BMDMs were pre-treated with 10 μM NVR compound, vehicle control (DMSO 0.5% v/v) or with the NFκB inhibitor Bay11-7082 (10 μM). After 15 minutes, Bay11-7082 was washed off and all cells were stimulated with 1 μg/mL LPS for 4 h. Cell lysates were probed for NLRP3 and pro-IL-1β induction. Western blots shown are representative of 3 biological repeats. **B** Cell treated as in (**A**). 4 hours post-LPS treatment cell culture supernatants were recovered and analysed for IL-6 content. Data correspond to 4 biological repeats. **C** COS7 cells were pre-treated with 10 μM NVR compound or vehicle control (DMSO 0.5% v/v) for 15 min and subsequently treated with 10 μM nigericin or vehicle control (EtOH 0.5%) for 90 min. Fixed cells were stained for Golgin97 (green), TGN46 (red) and nucleus (DAPI, blue). Images shown are representative of 4 biological repeats. Scale bar corresponds to 10 μm. **D** Pearson’s correlation coefficient (PCC) between Golgin97 and TGN46 for the cells treated in C. Data correspond to median +/− interquartile range from 4 biological repeats, where each datapoint is an individual field of view. Differences assessed by Kruskal-Wallis followed by Dunn’s post-test. ****p < 0.001. **E** LPS-primed BMDMs (1 μg/mL LPS; 4 h) were pre-treated with Cycloheximide (Chx, 10 μg/mL) or vehicle control (DMSO 0.5% v/v) for 30 min and subsequently stimulated with 10 μM nigericin in the presence of 10 μM NVR compounds or DMSO. After 90 min, culture supernatant was recovered and analysed for IL-1β content (left) or LDH release (right). Data correspond to mean +/− SD from 4 biological repeats. Differences assessed by two-way ANOVA followed by Dunnett’s post-test. ****p < 0.001. See also Fig. S4 for efficacy check of Chx.

We next set out to test whether squaramides affected the induction of NLRP3 or pro-IL-1β expression during the priming step in response to LPS. BMDMs were pre-treated with 10 μM NVR77/83 or vehicle for 15 min and subsequently primed with LPS for a further 4 h (Fig. 5). Pre-treatment of BMDMs with NVR77 or NVR83 prior to LPS did not change LPS-induced NLRP3 or pro-IL-1β expression (Fig. 5A), nor secretion of the pro-inflammatory cytokine IL-6 (Fig. 5B). As a control, treatment with the NFκB inhibitor Bay11-7082 completely inhibited LPS-induced NLRP3 and pro-IL-1β protein expression and IL-6 secretion (Fig. 5A, B).

Our group and others have recently described that NLRP3 inflammasome activators can cause dysfunction in the trafficking of endosomal cargoes [5, 6], which can be assessed as loss of co-localisation between the trans-Golgi network (TGN) marker TGN46 (that cycles to the plasma membrane and is trapped in endosomes when trafficking is disrupted [24, 25]) and the permanently resident TGN marker Golgin97 [26]. We tested whether NVR77 or NVR83 affected vesiculation of TGN46 in response to nigericin using COS7 cells, as have been used previously to observe this effect [5] (Fig. 5C, D). As expected, nigericin caused a significant loss of co-localisation between TGN46 and Golgin97 compared to vehicle control. However, neither NVR77 nor NVR83 affected TGN46 and Golgin97 co-localisation either on their own, or the reduced co-localisation elicited by nigericin (Fig. 5C, D), suggesting the squaramides exert their effects independently of endosomal cargo trafficking.

To determine whether the potentiating effects of squaramides NVR77 and NVR83 required new protein synthesis we performed inflammasome activation assays in the presence of the translation inhibitor cycloheximide (Chx, Fig. 5E, F). LPS-primed BMDMs were pre-treated with 10 μg/mL Chx or vehicle control for 30 min and subsequently treated with squaramides NVR77 and NVR83 or vehicle control for a further 15 min. The cells were then activated with 10 μM nigericin and the amount of IL-1β and LDH released was assessed after 90 min. Both NVR77 and NVR83 enhanced IL-1β secretion and LDH release in the presence of Chx (Fig. 5E, F). To validate the effectiveness of Chx as a translation inhibitor in the timeframe and concentration used, primary BMDMs were LPS-primed in the presence of 10 μg/mL Chx or vehicle control for 2 h (Fig. S4). In this setting and as expected, Chx was able to suppress induction of NLRP3 and pro-IL-1β expression (Fig. S4). These data suggest that the potentiating effects of the squaramide NVRs occurred independently of new protein synthesis.

The effect of the squaramide compounds on mitochondria was tested using MitoSOX, which measures mitochondrial superoxide production. BMDMs were loaded with MitoSOX for 10 min before being treated with 10 µM NVR77/83 in conjunction with 10 µM Nigericin or vehicle control (EtOH 0.5% v/v). Fluorescence intensity was recorded 2 h after treatment and showed that neither NVR77 nor NVR83 affected mitochondrial superoxide production (Fig. S5).

### NVR77 and NVR83 act by lowering intracellular K+

**Fig. 6:**
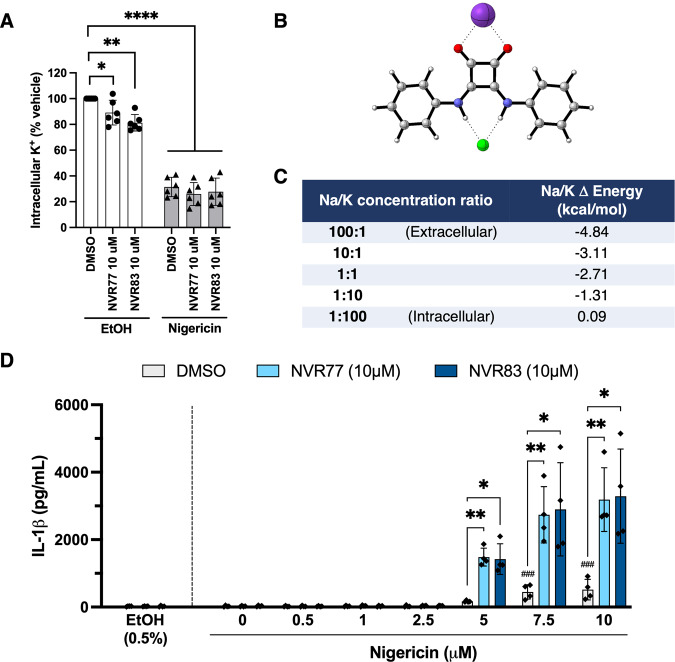
Squaramides decrease intracellular potassium concentration. **A** LPS-primed (1 μg/mL; 4 h) BMDMs were treated with 10 μM NVR compound or vehicle control (DMSO 0.5% v/v) in conjunction with 10 μM nigericin or vehicle control (EtOH 0.5% v/v) for 15 min. Intracellular potassium content was determined by ICP-MS from frozen cell pellets. Data correspond to 6 biological repeats. Differences to a theoretical mean of 100 were assessed by multiple *t*-tests and corrected for multiple comparisons with Holm-Sidak method. *p < 0.05, **p < 0.01, ****p < 0.0001. **B** Modelled structure of NVR77 in complex with potassium (purple) and chloride (green). See also Figs. S6 and S7, and Supplementary information for details of modelling parameters. **C** Table illustrating the binding energies for the NVR77:Chloride:Sodium complex when considering the relative abundance of sodium and potassium. **D** LPS-primed (1 μg/mL; 4 h) BMDMs were treated with 10 μM NVR compound or vehicle control (DMSO 0.5% v/v) for 15 min before addition of nigericin (1–10 μM) or vehicle control (EtOH 0.5% v/v). After 90 minutes, culture supernatant was recovered and assessed for IL-1β content by ELISA. Data correspond to mean +/− SD of 4 biological repeats. Differences assessed by Two-way ANOVA with Dunnett’s post test. *p < 0.05, **p < 0.01 Hashtags denote differences with vehicle control. ^###^*p* < 0.001.

Given that the squaramides were selectively potentiating K^+^ efflux-dependent NLRP3 inflammasome activation, we assessed their effect on intracellular K^+^ levels. LPS-primed BMDMs were treated with NVR77 or NVR83 in conjunction with 10 μM nigericin or vehicle control for 15 minutes. Intracellular K^+^ levels were assessed by inductively coupled plasma-mass spectrometry (ICP-MS) from frozen cell pellets. As expected, treatment with nigericin greatly decreased intracellular K^+^ levels, and this was not altered by the presence of the squaramide NVRs (Fig. 6A). In the absence of nigericin however, the squaramide NVRs significantly reduced the levels of intracellular K^+^ (Fig. 6A).

In an attempt to rationalise the squaramide compounds’ ability to reduce intracellular K^+^ levels, density functional calculations were conducted on NVR77 complexed with a range of anions, and the cations Na^+^ and K^+^ (see Supplementary information for full details). Complexation of NVR77 with anions alone showed a clear preference for Cl^−^, being the only anion for which the change in free energy suggests a preference for complex formation (exergonic, −2.25 kcal/mol, Table SI2). Complexes with other physiologically relevant anions (bicarbonate, phosphate, carboxylate, sulphate, nitrate) were all shown to be uphill in free energy terms (endergonic, Table SI2). In agreement with a number of squaramides in the Cambridge Structural Database [27] (see Table SI1) the two -NH groups in the squaramide complex to the Cl^−^ anion (Fig. 6B). These calculations support Cl^−^ being the physiological anion most likely to be found interacting with NVR77 in both intracellular and extracellular environments assuming the typical concentrations (Intracellular Cl^−^: 3–4 mEq/L and Extracellular Cl^−^: 98–106 mEq/L).

Formation of the complex of Cl^−^ anion with NVR77 and either Na^+^ or K^+^ from its three components was computed to be energetically more favourable than with Cl^−^ alone (Table SI2). The Na^+^ complex (−10.25 kcal/mol) was more stable than the K^+^ complex (−7.54 kcal/mol), with a 2.71 kcal/mol preference for Na^+^. Apart from the binding energies it is important to consider the relative concentrations of both cations to predict the complex most likely to be present. The extracellular medium for mammalian cells is typically high in Na^+^ and low in K^+^ with a 25–42:1 Na/K ratio (typical extracellular values Na^+^: 135–148 mEq/L and K^+^:3.5–5.4 mEq/L), therefore the NVR77:Cl^−^:Na^+^ complex is predicted to be dominant in the extracellular space. In contrast, the intracellular concentrations are reversed, with K^+^ being very high (140–150 mEq/L) when compared to Na^+^ (10–14 mEq/L) leading to a 1:10–150, Na:K ratio. Calculations show that a 10-fold decrease in the Na/K ratio decreases the difference by 1.4 kcal/mol and a 100-fold decrease by 2.8 kcal/mol (Fig. 6C). Therefore, at a 1:100 Na/K concentration, which is consistent with typical intracellular conditions, the K^+^ complex is modestly preferred.

Having seen a decrease of intracellular K^+^ upon treatment with NVR77/83 on their own, we explored whether the presence of the squaramides could lower the threshold for the K^+^ efflux-dependent activation of the NLRP3 inflammasome and enable IL-1β secretion in the presence of sub-optimal doses of nigericin. LPS-primed BMDMs were pre-treated with NVR77 or NVR83 or vehicle control for 15 min before being stimulated with increasing doses of nigericin (1–10 μM). After 90 minutes, the amount of IL-1β released was assessed by ELISA (Fig. 6D). The presence of NVR77 or NVR83 enabled significant IL-1β secretion when cells were stimulated with 5 μM of nigericin, a concentration of nigericin which did not elicit statistically significant IL-1β secretion on its own (Fig. 6D) suggesting the squaramides lowered the threshold of activation.

In summary, these calculations and data are consistent with NVR77 existing as the overall neutral Na^+^ Cl^−^ complex which can traverse the cell membrane. Once inside the cell, the high K^+^ concentrations cause a switch of Na^+^ to K^+^ in the NVR77:Cl^−^ complex (Fig. 6C). This complex is proposed to exit the cell, which lowers the intracellular K^+^ concentration, thus lowering the threshold for NLPR3 activation (Fig. 6D). In contrast to the squaramides with electron-withdrawing groups which are proposed to exchange anions, the cell permeable neutral or electron-donating squaramides NVR77/83 are proposed to lower the intracellular K^+^ levels by cation exchange.

## Discussion

Here we report small symmetrical squaramide compounds, NVR77 and NVR83, are enhancers of NLRP3 inflammasome activation. The squaramide compounds were unable to activate the inflammasome on their own and selectively increased IL-1β secretion induced by K^+^ efflux-dependent agonists. The literature on squaramide compounds describes their capacity to transport anions across lipid bilayers and alter intracellular Cl^−^ concentrations [13, 15]. NLRP3 inflammasome activation is very sensitive to ionic flux; activation can be triggered by K^+^-free buffers and conversely blocked by high extracellular K^+^ [7, 28]. In fact, K^+^ efflux was thought to be the common trigger event for most NLRP3 inflammasome agonists [7] until the report of imiquimod acting in a K^+^ efflux-independent mechanism [16]. With K^+^ efflux being accompanied by Cl^−^ efflux, the selective inflammasome enhancing abilities of the squaramide compounds here reported would fit with their proposed role of synthetic Cl^−^ transporters [15]. It should be noted, however, that the available literature on squaramide compounds does not include data on NVR77 or NVR83 specifically, and that squaramides with electron-withdrawing substituents in the ring, which neither NVR77 nor NVR83 possess, were most effective in their Cl^−^ transport abilities [13, 15]. Thus, we turned our attention to the possibility of these squaramides affecting K^+^ concentration. To-date there is no report of squaramide compounds acting as cation transporters. Computational analysis carried out on NVR77 showed that complexation of the NVR77 squaramide with Cl^−^ at the -NH groups and Na^+^ or K^+^ at the carbonyl group were the predicted predominant species. Interestingly, predicted binding energies for Ca^2+^ at the carbonyl groups with a number of physiologically relevant anions at the -NH groups were positive (unfavourable) (see Table SI2), highlighting the selective nature of the squaramides for Na^+^/K^+^. Furthermore, the stark difference in K^+^ concentration intra- and extra-cellularly allows for a change of preference for either cation depending on the location of the complex, exchanging K^+^ for Na^+^ when extracellularly and vice versa. This model supports our finding that treatment with NVR77 or NVR83 decreased intracellular K^+^ concentration and thus sensitised cells to sub-optimal doses of nigericin. The change in K^+^ concentration elicited by the squaramides was significant but mild in comparison to nigericin. This is reflected in the fact that treatment with the squaramide compounds alone was not sufficient to trigger activation of the NLRP3 inflammasome. Instead, the presence of the squaramides lowered the threshold for activation with other agonists such as nigericin. We did not observe a difference in K^+^ concentration in cells treated with the squaramides in the presence of nigericin despite observing an increase in IL-1β secretion.

Overall, this is the first report on the effects of squaramides on the inflammasome and of their ability to cause a movement of K^+^ from the cell. As enhancers of inflammation rather than inflammasome activators per se, one future use of squaramide-like compounds could be as adjuvants in the formulation of neoantigen anti-cancer vaccines, where enhanced immunogenicity is required [29]. Squaramides could also be used in the treatment of chronic infection where the immune response elicited is not sufficient to eliminate the threat, a treatment strategy that has shown promise for persistent skin infection [30]. Beyond this context, synthetic compounds able to lower intracellular K^+^ concentration could be of potential use in anti-cancer therapies, as there is evidence supporting an involvement of K^+^ efflux in apoptosis [31, 32]. Although more research is needed to dissect the mode of action of these compounds in different settings, it is a promising avenue to explore and highlights a potential future role of squaramide compounds as therapeutics in different scenarios.

## Materials and methods

### Mice

Wild-type C57BL/6 (Charles River, UK) or ASC-citrine C57BL/6 mice [18] were maintained in house for generation of primary bone marrow-derived macrophage cultures. All procedures were carried out in accordance with the Home Office (Animals) Scientific Procedures Act (1986).

### Generation of bone marrow-derived macrophages (BMDMs)

Mice were euthanized and the bone marrow from femur and tibia bones was isolated. Red blood cells were lysed with ACK lysis buffer (Fisher Scientific) and the remaining cells were cultured in L929-containing Dulbecco’s Modified Eagle’s Medium (DMEM) supplemented with foetal bovine serum (10% v/v, FBS, Life Technologies), 100 U/mL penicillin and 100 mg/mL streptomycin (1% v/v, P/S, Sigma). After 6–7 days in culture cells were harvested and seeded overnight at a density of 1 × 10^6^ cell/mL in DMEM supplemented with 10% v/v FBS and 1% v/v P/S (henceforth cDMEM) in 96-well plates unless otherwise stated.

### Inflammasome activation assays

BMDMs were primed with LPS (1 μg/mL) in cDMEM for 4 h. Cells were then pre-treated with the squaramide NVR compound (10 μM) or vehicle control (DMSO 0.5% v/v) in serum-free DMEM. After 15 min, nigericin (10 μM), imiquimod (75 μM), ATP (5 mM), LLOMe (1 mM) or vehicle control (EtOH 0.5 % v/v) was added into the well. For time course experiments, squaramide compounds (NVR77/83, 10 μM) were added 15 min prior, in conjunction with, or 15 or 30 min after addition of nigericin (10 μM). For cycloheximide experiments, LPS-primed BMDMs were pre-treated with cycloheximide (Chx, 10 μg/mL, Sigma) or vehicle control (DMSO 0.5% v/v) in serum-free DMEM. After 30 min, cells were further treated with the squaramide compounds NVR77/83 (10 μM) or vehicle control (DMSO 0.5% v/v) in conjunction with nigericin (10 μM) or vehicle control (EtOH 0.5% v/v). To confirm efficacy of Chx treatment a priming experiment was performed (see below). After 90 min with the inflammasome activator cells were placed on ice, supernatant recovered, and cells lysed for future analysis.

For NLRC4 or AIM2 inflammasome activation assays, LPS-primed (1 μg/mL; 4 h) BMDMs were treated with NVR77/83 (10 μM) or vehicle control (DMSO 0.5% v/v) in serum-free DMEM. After 15 min, cells were transfected with flagellin (1 μg/mL, Sigma), polydAdT (1 μg/mL, Invivogen) or mock (PBS) using Lipofectamine 3000 (Invitrogen) according to the manufacturers’ instructions. After 3 h cells were placed on ice and supernatant recovered for future assays.

### Priming experiments

BMDMs were pre-treated with the squaramides NVR77/83 (10 μM) or vehicle control (DMSO 0.5% v/v) or Bay11-7082 (10 μM, ApexBio A4210, USA, Houston) or Chx (10 μg/mL). After 15 min, Bay11-7082 was removed and replaced with fresh cDMEM and all wells were treated with LPS (1 μg/mL). After 4 h, or 2 h for Chx experiments, cells were placed on ice, supernatant recovered, and cells lysed for future analysis.

### ASC oligomerisation assay

BMDMs were seeded at a density of 1 × 10^6^ cells/mL onto 12-well plates in cDMEM and incubated at 37 °C and 5% CO_2_ overnight. The following day, cells were treated as described in inflammasome activation assays above. At endpoint, cells were lysed by addition of 1% v/v Triton X-100 and protease inhibitor cocktail (Merck Millipore, 539131). Cell lysates were spun at 6800 × *g* for 20 min at 4 °C and the Triton X-100 soluble and insoluble fractions were obtained. The insoluble fraction was DSS-crosslinked (2 mM, Thermo) for 30 min at RT, spun at 6800 × *g* for 20 min and eluted in Laemmli buffer for further analysis. Both soluble and DSS-crosslinked-insoluble fractions were used for immunoblotting.

### Live-cell imaging

ASC-citrine primary BMDMs were seeded at a density of 1 × 10^6^ cells/mL onto black-walled 96-well plates in cDMEM and incubated at 37 °C and 5% CO_2_ overnight. The following day, cells were LPS-primed (1 μg/mL) in cDMEM. After 4 h, the medium was replaced with OptiMEM containing the squaramide compounds (NVR77/83, 10 μM) or vehicle control (DMSO 0.5% v/v) in addition to the inflammasome activator (nigericin (10 μM) or imiquimod (75 μM)) or vehicle control (EtOH 0.5% v/v). Cells were imaged every 15 min over a period of 3 h on an Incucyte S3 Live Cell Analysis system (Essen Bioscience, Germany) using a 20x/0.61 S Plan Fluor objective and the green filter set for fluorescent images. Cells were kept at 37 °C and 5% CO_2_ throughout the experiment. ASC speck number was assessed manually for each treatment.

### TGN vesiculation assay and imaging

COS7 cells (ATCC) were seeded at a density of 1 × 10^6^ cells/mL onto 24-well plates containing 13 mm glass coverslips and incubated in cDMEM overnight. Cells were then pre-treated with the squaramide NVR compound (NVR77/83, 10 μM) or vehicle control (DMSO 0.5% v/v) in serum-free DMEM for 15 min before being further stimulated with nigericin (10 μM) or vehicle control (EtOH 0.5% v/v). After 90 min cells were washed 3 times with sterile PBS and fixed by addition of paraformaldehyde solution (4% w/v, Sigma) for 20 min at RT. Cells were then permeabilised with 0.1% Triton X-100 (PBS-Triton, Sigma) in PBS for 5 min and blocked with 5% BSA in PBS-Triton for 30 min at RT. Coverslips were incubated with sheep anti-human TGN46 (Biorad, AHP500G) and rabbit anti-human Golgin97 (Abcam, ab84340) in PBS-Triton for 1 h at RT. Following 3 washes with PBS-Triton, coverslips were incubated with appropriate secondary antibodies (Invitrogen) for 1 h at RT. Nuclei were stained with DAPI (Invitrogen, D1306). Coverslips were washed with PBS-Triton followed by distilled water before being carefully mounted onto glass slides using ProLong Gold antifade mounting reagent (Invitrogen) and left to dry overnight at RT. Slides were imaged on a Leica TCS SP8 AOBS inverted confocal using a 40x/HC PL APO CS2 objective. The confocal settings were as follows: pinhole 1 airy unit, scan speed 600 Hz bidirectional, format 1024 × 1024. The confocal software (LAS X v3.5.1.18803) was used to determine the optimal number of Z sections for optical stacks.

Resulting Z-stacks were analysed using Fiji (ImageJ) software. Pearson’s correlation co-efficient (PCC) between TGN46 and Golgin97 for each field of view was calculated using the Coloc 2 plugin on the maximum intensity projection of the Z-stacks.

### Mitochondrial superoxide production assay

BMDMs were seeded at a density of 1 × 10^6^ cells/mL onto black-walled 96-well plates in cDMEM and incubated at 37 °C and 5% CO_2_ overnight. The following day, cells were loaded with 5 µM MitoSOX (Invitrogen, M36008) diluted in PBS. After 10 min, cells were washed three times with OpitMEM and treated with 10 µM Nigericin or vehicle control (EtOH 0.5% v/v) in conjunction with 10 µM NVR77/83 or DMSO control (0.5% v/v). Treatment with 1% H_2_O_2_ was used as a positive control. Cells were imaged on an Incucyte S3 Live Cell Analysis system (Essen Bioscience, Germany) using a 20x/0.61 S Plan Fluor objective and the red filter set for fluorescent images. Cells were kept at 37 °C and 5% CO_2_ throughout the experiment.

### ELISA

Supernatants were analysed for IL-1β (DY401) or IL-6 (DY406) content using duo-set ELISA kits from R&D Systems according to the manufacturer’s instructions.

### Cell death assays

Cell death was assessed by measuring release of lactate dehydrogenase (LDH) into the supernatant of cultured cells using CytoTox 96 Non-Radioactive Cytotoxicity Assay (Promega) according to the manufacturer’s instructions.

### Western blotting

Samples were run on SDS PAGE and transferred onto PVDF membranes using a semidry Trans-Blot Turbo system (Bio-Rad). Membranes were blocked for 1 h at RT in 5% w/v BSA in PBS 0.1% v/v Tween-20 (PBST) before being incubated overnight at 4 °C with one of the following primary antibodies in 1% BSA in PBST: goat anti-mouse IL-1β (R&D Systems, AF-401-NA, UK), mouse anti-mouse NLRP3 (Adipogen, G-20B-0014-C100, Switzerland), rabbit anti-mouse caspase-1 (Abcam, ab179515, UK), rabbit anti-mouse ASC (Cell Signalling Technologies, 67824, UK). Membranes were washed 3 times with PBST before being incubated for 1 h at RT with the appropriate HRP-conjugated secondary antibody (Dako, UK) or mouse anti-β-actin-HRP antibody (Sigma, A3854). After a further 3 washes with PBST, target proteins were visualised by chemiluminescence using Amersham ECL prime detection reagent (GE healthcare, UK) and G:Box Chemi XX6 (Syngene, UK). Uncropped western blot images can be found in Supplementary Information.

### Intracellular potassium determination

BMDMs were seeded at a density of 1 × 10^6^ cells/mL onto 6-well plates in cDMEM and incubated at 37 °C and 5% CO_2_ overnight. The following day, cells were primed with LPS (1 μg/mL) in cDMEM for 4 h. During the last hour of priming the caspase1 inhibitor Ac-YVAD-cmk (50 μM, Invivogen) was added to prevent pyroptosis. BMDMs were then treated with 10 μM NVR compound or vehicle control (DMSO 0.5% v/v) in conjunction with 10 μM nigericin or vehicle control (EtOH 0.5% v/v) in serum-free DMEM. After 15 min, cells were washed in a K^+^/Cl^−^ free buffer (130 mM NaGluconate, 7 mM Na_2_HPO_4_, 3 mM NaH_2_PO_4_), spun down, weighed, and frozen. Intracellular K^+^ content was determined by inductively coupled plasma-mass spectrometry (ICP-MS) from the frozen cell pellets using a 7700x Inductively Coupled Plasma-Mass Spectrometer (Agilent) at the Centre for Advanced Discovery and Experimental Therapeutics (CADET, University of Manchester). Intracellular K^+^ measurements were corrected against cell pellet weight prior to ICP-MS processing.

### Computational analysis

The squaramide NVR77 in complexation with different anions and cations was modelled. Calculations were performed at the M06/6-31 + G** level incorporating solvation via the IEFPCM with settings appropriate to water in Gaussian16 [33–40]. Free energies at 1 M concentration and 310 K were computed using Goodvibes v.3.0.1 [41]. See supplementary information for more details.

### Statistical analysis

Data were analysed using GraphPad v9. Normally distributed data (as determined by Shapiro-Wilk normality test) were analysed by two-way analysis of variance (ANOVA) followed by Dunnett’s post-test. Non-parametric data were analysed by Kruskal-Wallis test followed by Dunn’s post-test. Normalised percentage data were analysed by multiple *t* tests compared to a value of 100% and corrected for multiple comparisons by Holm-Sidak post-test. In all cases *p < 0.05, **p < 0.01, ***p < 0.001, ****p < 0.0001.

### Supplementary information

Supplementary Information

Uncropped Western Blots

Computational modelling _ raw data

## Data Availability

All data will be made available upon reasonable request.

## References

[1] Seoane PI, Lee B, Hoyle C, Yu S, Lopez-Castejon G, Lowe M (2020). The NLRP3-inflammasome as a sensor of organelle dysfunction. J Cell Biol.

[2] Evavold CL, Kagan JC (2022). Diverse control mechanisms of the interleukin-1 cytokine family. Front Cell Dev Biol.

[3] Mariathasan S, Weiss DS, Newton K, McBride J, O’Rourke K, Roose-Girma M (2006). Cryopyrin activates the inflammasome in response to toxins and ATP. Nature.

[4] Hornung V, Bauernfeind F, Halle A, Samstad EO, Kono H, Rock KL (2008). Silica crystals and aluminum salts activate the NALP3 inflammasome through phagosomal destabilization. Nat Immunol.

[5] Lee B, Hoyle C, Wellens R, Green JP, Martin-Sanchez F, Williams DM (2023). Disruptions in endocytic traffic contribute to the activation of the NLRP3 inflammasome. Sci Signal.

[6] Zhang Z, Venditti R, Ran L, Liu Z, Vivot K, Schurmann A (2023). Distinct changes in endosomal composition promote NLRP3 inflammasome activation. Nat Immunol.

[7] Munoz-Planillo R, Kuffa P, Martinez-Colon G, Smith BL, Rajendiran TM, Nunez GK (2013). (+) efflux is the common trigger of NLRP3 inflammasome activation by bacterial toxins and particulate matter. Immunity.

[8] Green JP, Yu S, Martin-Sanchez F, Pelegrin P, Lopez-Castejon G, Lawrence CB (2018). Chloride regulates dynamic NLRP3-dependent ASC oligomerization and inflammasome priming. Proc Natl Acad Sci USA.

[9] Wang L, Hauenstein AV (2020). The NLRP3 inflammasome: mechanism of action, role in disease and therapies. Mol Aspects Med.

[10] Hu Z, Wu D, Lu J, Zhang Y, Yu SM, Xie Y (2022). Inflammasome activation dampens type i ifn signaling to strengthen anti-toxoplasma immunity. mBio.

[11] Karki R, Man SM, Malireddi RKS, Gurung P, Vogel P, Lamkanfi M (2015). Concerted activation of the AIM2 and NLRP3 inflammasomes orchestrates host protection against Aspergillus infection. Cell Host Microbe.

[12] Swanton T, Beswick JA, Hammadi H, Morris L, Williams D, de Cesco S (2020). Selective inhibition of the K(+) efflux sensitive NLRP3 pathway by Cl(-) channel modulation. Chem Sci.

[13] Busschaert N, Kirby IL, Young S, Coles SJ, Horton PN, Light ME (2012). Squaramides as potent transmembrane anion transporters. Angew Chem Int Ed Engl.

[14] Marques I, Costa PMR, Miranda Q, Busschaert M, Howe N, Clarke ENW (2018). Full elucidation of the transmembrane anion transport mechanism of squaramides using in silico investigations. Phys Chem Chem Phys.

[15] Busschaert N, Park SH, Baek KH, Choi YP, Park J, Howe ENW (2017). A synthetic ion transporter that disrupts autophagy and induces apoptosis by perturbing cellular chloride concentrations. Nat Chem.

[16] Gross CJ, Mishra R, Schneider KS, Medard G, Wettmarshausen J, Dittlein DC (2016). K(+) Efflux-independent NLRP3 inflammasome activation by small molecules targeting mitochondria. Immunity.

[17] Coll RC, Robertson AA, Chae JJ, Higgins SC, Munoz-Planillo R, Inserra MC (2015). A small-molecule inhibitor of the NLRP3 inflammasome for the treatment of inflammatory diseases. Nat Med.

[18] Tzeng TC, Schattgen S, Monks B, Wang D, Cerny A, Latz E (2016). A fluorescent reporter mouse for inflammasome assembly demonstrates an important role for cell-bound and free ASC specks during in vivo infection. Cell Rep.

[19] Hoyle C, Green JP, Allan SM, Brough D, Lemarchand E (2022). Itaconate and fumarate derivatives inhibit priming and activation of the canonical NLRP3 inflammasome in macrophages. Immunology.

[20] Franchi L, Amer A, Body-Malapel M, Kanneganti TD, Ozoren N, Jagirdar R (2006). Cytosolic flagellin requires Ipaf for activation of caspase-1 and interleukin 1beta in salmonella-infected macrophages. Nat Immunol.

[21] Miao EA, Alpuche-Aranda CM, Dors M, Clark AE, Bader MW, Miller SI (2006). Cytoplasmic flagellin activates caspase-1 and secretion of interleukin 1beta via Ipaf. Nat Immunol.

[22] Fernandes-Alnemri T, Yu JW, Datta P, Wu J, Alnemri ES (2009). AIM2 activates the inflammasome and cell death in response to cytoplasmic DNA. Nature.

[23] Hornung V, Ablasser A, Charrel-Dennis M, Bauernfeind F, Horvath G, Caffrey DR (2009). AIM2 recognizes cytosolic dsDNA and forms a caspase-1-activating inflammasome with ASC. Nature.

[24] Chapman RE, Munro S (1994). Retrieval of TGN proteins from the cell surface requires endosomal acidification. EMBO J.

[25] Reaves B, Banting G (1994). Vacuolar ATPase inactivation blocks recycling to the trans-Golgi network from the plasma membrane. FEBS Lett.

[26] Griffith KJ, Chan EK, Lung CC, Hamel JC, Guo X, Miyachi K (1997). Molecular cloning of a novel 97-kd Golgi complex autoantigen associated with Sjogren’s syndrome. Arthritis Rheum.

[27] CCDC. Cambridge Structural Database. https://www.ccdc.cam.ac.uk/.

[28] Petrilli V, Papin S, Dostert C, Mayor A, Martinon F, Tschopp J (2007). Activation of the NALP3 inflammasome is triggered by low intracellular potassium concentration. Cell Death Differ.

[29] Manna S, Maiti S, Shen J, Weiss A, Mulder E, Du W (2023). Nanovaccine that activates the NLRP3 inflammasome enhances tumor specific activation of anti-cancer immunity. Biomaterials.

[30] Sousa Mda G, Reid DM, Schweighoffer E, Tybulewicz V, Ruland J, Langhorne J (2011). Restoration of pattern recognition receptor costimulation to treat chromoblastomycosis, a chronic fungal infection of the skin. Cell Host Microbe.

[31] Bortner CD, Hughes FM, Cidlowski JA (1997). A primary role for K+ and Na+ efflux in the activation of apoptosis. J Biol Chem.

[32] Remillard CV, Yuan JX (2004). Activation of K+ channels: an essential pathway in programmed cell death. Am J Physiol Lung Cell Mol Physiol.

[33] Frisch MJ, Trucks GW, Schlegel HB, Scuseria GE, Robb MA, Cheeseman JR, et al. Gaussian 16 Rev. C.01. Wallingford, CT, 2016.

[34] Hariharan PC, Pople JA (1973). The influence of polarization functions on molecular orbital hydrogenation energies. Theoretica Chimica Acta.

[35] Hehre WJ, Ditchfield R, Pople JA (1972). Self—consistent molecular orbital methods. XII. Further extensions of Gaussian—type basis sets for use in molecular orbital studies of organic molecules. J Chem Phys.

[36] Krishnan R, Binkley JS, Seeger R, Pople JA (1980). Self‐consistent molecular orbital methods. XX. A basis set for correlated wave functions. J Chem Phys.

[37] Tomasi J, Mennucci B, Cammi R (2005). Quantum mechanical continuum solvation models. Chem Rev.

[38] Zhao Y, Truhlar DG (2006). A new local density functional for main-group thermochemistry, transition metal bonding, thermochemical kinetics, and noncovalent interactions. J Chem Phys.

[39] Zhao Y, Truhlar DG (2008). Density functionals with broad applicability in chemistry. Acc Chem Res.

[40] Zhao Y, Truhlar DG (2008). The M06 suite of density functionals for main group thermochemistry, thermochemical kinetics, noncovalent interactions, excited states, and transition elements: two new functionals and systematic testing of four M06-class functionals and 12 other functionals. Theor Chem Acc.

[41] Ignacio Funes-Ardoiz RSP. GoodVibes. version 2.0.3 (v2.0.3) ed. Zenodo 2018.

