# Supplementary material for: Squaramides enhance NLRP3 inflammasome activation by lowering intracellular potassium
Source: Cell Death Discov. 2023 Dec 22;9:469. doi: 10.1038/s41420-023-01756-9 (PMC10739973; doi:10.1038/s41420-023-01756-9)
Supplement: Supplementary file 1 — Supplementary Information [file 41420_2023_1756_MOESM1_ESM.docx]

## Supplementary Information

Contents

1. Chemistry synthesis (pages 1 – 5)
2. Biological supplementary figures (pages 6 – 9)
3. Computational analysis (pages 9 – 16)
4. References (pages 16 – 17)

## 1. Synthesis of squaramide compounds NVR77-84

**1.1 Methods and Instrumentation**

All chemicals, solvents and deuterated solvents were purchased from Sigma-Aldrich, Alfa-Aesar, Fluorochem or Fisher Scientific. ^1^H, ^13^C, and ^19^F NMR spectra were recorded on a Bruker Avance 400 or 300 MHz spectrometer. Chemical shifts (δ) are defined in parts per million (ppm). ^1^H NMR spectra were referenced to residual undeuterated solvent (CDCl_3_, δ = 7.27 ppm; DMSO-d_6_, δ = 2.50 ppm). ^13^C NMR spectra were referenced to residual undeuterated solvent (CDCl_3_, δ = 77.16 ppm, DMSO-d_6_ = 39.51) as an internal reference. Accurate mass determination was carried out on a Thermo Exactive™ Plus EMR Orbitrap™ LC-MS system. Molecular ion peaks are defined as mass/charge (m/z) ratios. Infrared spectroscopy was recorded on a Bruker ALPHA II FTIR Spectrometer and processed using the OPUS software package. Analytical thin-layer chromatography (TLC) was performed using silica gel 60 on aluminium sheets coated with F254 indicator. All spots were visualised with KMnO_4_ or ultraviolet light using a MV Mineralight lamp (254/365) UVGL-58. Flash column chromatography was performed using silica gel with particle size 40-63 μm. Evaporation of solvents was conducted on a Buchi Rotavapor R-200.

LC data was obtained using a Waters ACQUITY UPLC PDA detector scanning between 210-400 nm. Mass spectrometry data was acquired using a Waters ACQUITY QDa detector scanning in the positive (ES^+^) and negative (ES^–^) modes between *m/z* 100-1000. Separation of components was achieved using a Waters ACQUITY UPLC BEH C18 1.7 µm 2.1 × 50 mm column coupled to a Waters ACQUTY UPLC BEH C18 1.7 µm VanGuard pre-column 2.1 × 5 mm. Columns were maintained at 40 °C throughout acquisition. The table below details a schematic for the standard four-minute run on the instrument using a flow rate of 0.60 mL min^−1^:

| Start Time / min | End Time / min | H_2_O : MeCN (%v/v) |
| --- | --- | --- |
| 0 | 0.5 | 95 : 5 |
| 0.5 | 2.5 | 95 : 5 → 5 : 95 |
| 2.5 | 3 | 5 : 95 |
| 3 | 3.1 | 5 : 95 → 95 : 5 |
| 3.1 | 4 | 95 : 5 |

All solvents obtained were of LC-MS grade (Fisher Optima) and were modified by the addition of 0.1% v/v formic acid (Fisher Optima). Samples were prepared in MeOH (Fisher Optima) and a 2.0 µL aliquot was extracted from each sample for analysis. Samples were maintained at 10 °C prior to acquisition. Solvents were combined using a Waters ACQUITY UPLC H-Class Quaternary Solvent Manager (QSM) with in-built degasser. Inert gas was provided using a Genius NM32LA nitrogen generator. The switch method consisted of 95:5 H_2_O:MeCN (with appropriate acidic or basic modifiers) running at 0.60 mL min^−1^ for two minutes immediately prior to acquisition. Data were processed using MassLynx V4.15.

### 1.2 General Synthesis for squaramide NVR compounds

The appropriate amine was added dropwise to a stirred solution of 3,4-diethoxy-3-cyclobutene-1,2-dione (0.26 mL, 1.8 mmol, 1 eq), zinc trifluoromethanesulfonate (128 mg, 0.35 mmol, 0.2 eq) and ethanol (25 mL). The reaction mixture was stirred for 16 hours at either 25°C or 80 °C. Upon cooling to room temperature, the reaction mixture was filtered and the precipitate washed with minimum ethanol, before being dried to yield the squaramide product.

3,4-Bis(phenylamino)cyclobut-3-ene-1,2-dione (NVR77)

General synthesis using aniline (0.35 mL, 3.9 mmol, 2.2 eq) at 25°C to yield a white solid (460 mg, 99%). ^1^H NMR (400 MHz, *DMSO-d*_6_) δ ppm 9.88 (br s, 2H, 2 × NH), 7.50 (br d, ^3^*J*_HH_ *=* 7.5 Hz, 4H, H2 + H6), 7.39 (br t, ^3^*J*_HH_ *=* 7.6 Hz, 4H, H3 + H5), 7.09 (br t, ^3^*J*_HH_ *=* 7.4 Hz, 2H, H4). ^13^C NMR (101 MHz, *DMSO-d*_6_) δ ppm 181.6 (s, 2 × C=O), 165.6 (s, 2 × C=C), 138.5 (s, C1), 129.4 (s, C3 + C5), 123.3 (s, C4), 118.5 (s, C2 + C6); IR: 3194, 3143, 3085, 3036, 3007, 2956, 2912, 2861, 1797, 1669, 1601, 1540 cm^−1^. LCMS (m/z): [M+H, 100%]^+^, 265.2; RT = 2.21 min; HRMS(HESI+) *(m/z)*: [M+H]^+^ calcd. for C_16_H_13_O_2_N_2_, 265.0972; found, 265.0970, error: 0.6 ppm. ^1^H and ^13^C NMR data corresponds to the literature (1, 2).

3,4-Bis((3-(trifluoromethyl)phenyl)amino)cyclobut-3-ene-1,2-dione (NVR78)

General synthesis using 3-trifluoromethylaniline (0.48 mL, 3.9 mmol, 2.2 eq) at 80 °C to yield a white solid (692 mg, 98%). ^1^H NMR (400 MHz, *DMSO-d*_6_) δ ppm 10.17 (br s, 2H, 2 × NH), 7.90 (br s, 2H, H2), 7.56 - 7.65 (m, 4H, H4, H5), 7.41 (br d, ^3^*J*_HH_ *=* 6.8 Hz, 2H, H6); ^13^C NMR (101 MHz, *DMSO-d*_6_) δ ppm 182.4 (s, 2 × C=O), 165.8 (s, 2 × C=C), 139.3 (s, C1), 130.6 (s, C5), 130.2 (q, *J =* 32.3 Hz, C3), 124.0 (q, ^1^*J*_CF_ *=* 271.9 Hz, 2 × CF_3_), 122.3 (s, C6), 119.6 (q, ^3^*J*_CF_ *=* 3.6 Hz, C4), 115.1 (q, ^3^*J*_CF_ *=* 3.9 Hz, C2); ^19^F NMR (376 MHz, *DMSO-d*_6_) δ ppm −61.4 (s, 2 × CF_3_); IR: 3353, 2991, 1782, 1663, 1634, 1593, 1564, 1402 cm^−1^. LCMS (m/z): [M+H, 100%]^+^, 401.2; RT = 2.62 min; HRMS(HESI+) *(m/z)*: [M+H]^+^ calcd. for C_18_H_10_O_2_N_2_F_6_Na, 423.0539; found, 423.0537, error: 0.4 ppm.

3,4-Bis((3-(trifluoromethoxy)phenyl)amino)cyclobut-3-ene-1,2-dione (NVR79)

General synthesis using 3-trifluoromethoxyaniline (0.52 mL, 3.9 mmol, 2.2 eq) at 80 °C to yield a pale-yellow solid (485 mg, 64%). ^1^H NMR (400 MHz, *DMSO-d*_6_) δ ppm 10.09 (s, 2H, 2 × NH), 7.63 (br s, 2H, H2), 7.50 (t, ^3^*J*_HH_ *=* 8.3 Hz, 2H, H5), 7.37 (br dd, ^3^*J*_HH_ *=* 8.3 Hz, ^4^*J*_HH_ = 1.5 Hz, 2H, H6), 7.07 (br d, ^3^*J*_HH_ *=* 8.3 Hz, 2H, H4); ^13^C NMR (101 MHz, *DMSO-d*_6_) δ ppm 182.1 (s, 2 × C=O), 165.7 (s, 2 × C=C), 149.1 (br q, ^3^*J*_CF_ *=* 2.0 Hz, C3), 140.2 (s, C1), 131.2 (s, C5), 120.1 (q, ^1^*J*_CF_ *=* 256.3 Hz, 2 × OCF_3_), 117.4 (s, C4), 115.2 (s, C6), 111.1 (s, C2); ^19^F NMR (376 MHz, *DMSO-d*_6_) δ ppm −56.7 (s, 2 × OCF_3_); IR: 3394, 3188, 3028, 2997, 1782, 1665, 1612, 1556, 1507 cm^−1^. LCMS (m/z): [M+H, 100%]^+^, 433.2; RT = 2.72 min; HRMS(HESI−) *(m/z)*: [M−H]^−^ calcd. for C_18_H_9_O_4_N_2_F_6_, 431.0461; found, 431.0465, error: 0.9 ppm.

3,4-Bis((3,5-bis(trifluoromethyl)phenyl)amino)cyclobut-3-ene-1,2-dione (NVR80)

General synthesis using 3,5-bis(trifluoromethyl)aniline (0.83 mL, 5.3 mmol, 3 eq) at 80 °C to yield a white solid (861 mg, 91%). ^1^H NMR (400 MHz, *DMSO-d*_6_) δ ppm 10.60 (br s, 2H, 2 × NH), 7.88 (br s, 4H, H2 + H6), 7.69 (br s, 2H, H4); ^13^C NMR (101 MHz, *DMSO-d*_6_) δ ppm 184.2 (s, 2 × C=O), 165.7 (s, 2 × C=C), 140.5 (s, C1), 131.1 (q, ^2^*J*_CF_ *=* 33.3 Hz, C3 + C5), 123.0 (q, ^1^*J*_CF_ *=* 274.2 Hz, 4 × CF_3_), 119.2 (s, C2 + C6), 116.0 (s, C4); ^19^F NMR (376 MHz, *DMSO-d*_6_) δ ppm −61.8 (s, 4 × CF_3_); IR: 3139, 2968, 2925, 1801, 1677, 1560, 1502 cm^−1^. LCMS (m/z): [M+H, 100%]^+^, 537.1; RT = 2.89 min; HRMS(HESI+) *(m/z)*: [M+Na]^+^ calcd. for C_20_H_8_O_2_N_2_F_12_Na, 559.0286; found, 559.0272, error: 2.6 ppm. ^1^H and ^13^C NMR data corresponds to the literature (1, 2).

**3,4-Bis(cyclohexylamino)cyclobut-3-ene-1,2-dione (NVR81)**

General synthesis using cyclohexylamine (0.443 mL, 3.9 mmol, 2.2 eq) at 25 °C to yield a white solid (470 mg, 97%). ^1^H NMR (400 MHz, *DMSO-d*_6_) δ ppm 7.31 (br s, 2H, 2 × NH), 3.77 (br s, 2H, H1), 1.82 - 1.95 (m, 4H, H2 + H6), 1.61 - 1.75 (m, 4H, H2 + H6), 1.55 (br d, ^2^*J*_HH_ *=* 12.0 Hz, 2H, H4), 1.10 - 1.38 (m, 10 H, H3 × 2, H4, H5 × 2); ^13^C NMR (101 MHz, *DMSO-d*_6_) δ ppm 181.9 (s, 2 × C=O), 167.0 (s, 2 × C=C), 52.0 (s, C1), 33.7 (s, C2 + C6), 24.8 (s, C3 + C5), 24.0 (s, C4); IR: 3145, 2929, 2851, 1797, 1640, 1544 cm^−1^. LCMS (m/z): [M+H, 100%]^+^, 277.3; RT = 2.26 min; HRMS(HESI−) *(m/z)*: [M−H]^−^ calcd. for C_16_H_24_O_2_N_2_Na, 299.1730; found, 299.1719, error: 3.7 ppm. ^1^H and ^13^C NMR data corresponds to the literature (3).

3,4-Bis((3,5-dimethylphenyl)amino)cyclobut-3-ene-1,2-dione (NVR82)

General synthesis using 3,5-dimethylaniline (0.66 mL, 5.3 mmol, 3 eq) at 80 °C to yield an off-white solid (555 mg, 98%). ^1^H NMR (400 MHz, *DMSO-d*_6_) δ ppm 9.73 (s, 2H, 2 × NH), 7.08 (s, 4H, H2 + H6), 6.72 (s, 2H, H4), 2.26 (s, 12H, 2 × CH_3_); ^13^C NMR (101 MHz, *DMSO-d*_6_) δ ppm 181.6 (s, 2 × C=O), 165.6 (s, 2 × C=C), 138.5 (s, C3 + C5), 138.4 (s, C1), 124.9 (s, C4), 116.3 (s, C2 + C6), 21.1 (s, 4 × CH_3_); IR: 3396, 3283, 3248, 3194, 3153, 3063, 2919, 1787, 1655, 1634, 1624, 1593, 1562, 1505 cm^−1^. LCMS (m/z): [M+H, 100%]^+^, 321.2; RT = 2.59 min; HRMS(HESI+) *(m/z)*: [M+Na]^+^ calcd. for C_20_H_20_O_2_N_2_Na, 343.1417; found, 343.1405, error: 3.5 ppm.

3,4-Bis((4-methoxyphenyl)amino)cyclobut-3-ene-1,2-dione (NVR83)

General synthesis using 4-methoxyaniline (477 mg, 3.9 mmol, 2.2 eq) at 80 °C to yield a beige solid (558 mg, 98%). ^1^H NMR (400 MHz, *DMSO-d*_6_) δ ppm 9.68 (s, 2H, 2 × NH), 7.40 (br d, *J =* 8.8 Hz, 4H, H2 + H6), 6.95 (br d, ^3^*J*_HH_ *=* 8.8 Hz, 4H, H3 + H5), 3.75 (s, 6H, 2 × OCH_3_); ^13^C NMR (101 MHz, *DMSO-d*_6_) δ ppm 181.2 (s, 2 × C=O), 165.0 (s, 2 × C=C), 155.58 (s, C4), 131.8 (s, C1), 120.0 (s, C2 + C6), 114.5 (s, C3 + C5), 55.3 (s, 2 × OCH_3_); IR: 2974, 1799, 1661, 1614, 1546, 1509 cm^−1^. LCMS (m/z): [M+H, 100%]^+^, 325.2; RT = 2.17 min; HRMS(HESI+) *(m/z)*: [M+Na]^+^ calcd. for C_18_H_16_O_4_N_2_Na, 347.1002; found, 347.0998, error: 1.2 ppm. ^1^H, ^13^C NMR and IR data corresponds to the literature (1).

3,4-Bis((4-fluorophenyl)amino)cyclobut-3-ene-1,2-dione (NVR84)

General synthesis using 4-fluoroaniline (430 mg, 3.9 mmol, 2.2 eq) at 80 °C to yield a pale yellow solid (511 mg, 97%). ^1^H NMR (400 MHz, *DMSO-d*_6_) δ ppm 9.85 (s, 2H, 2 × NH), 7.47 (br dd, ^3^*J*_HH_ *=* 9.0 Hz, ^4^*J*_HF_ = 4.5 Hz, 4H, H2 + H6), 7.22 (br t, ^3^*J*_HH_ ≈ ^3^*J*_HF_ = 8.8 Hz, 4H, H3, H5); ^13^C NMR (101 MHz, *DMSO-d*_6_) δ ppm 181.7 (s, 2 × C=O), 165.4 (s, 2 × C=C), 158.5 (d, ^1^*J*_CF_ *=* 239.6 Hz, 2 × CF), 135.0 (d, ^4^*J*_CF_ *=* 2.9 Hz, C1"), 120.5 (d, ^3^*J*_CF_ *=* 7.8 Hz, C2 + C6), 116.0 (d, ^2^*J*_CF_ *=* 22.5 Hz, C3 + C5); ^19^F NMR (376 MHz, *DMSO-d*_6_) δ ppm −119.9 (tt, ^3^*J*_HF_ *=* 8.8 Hz, ^4^*J*_HF_ = 4.5 Hz, 2 × CF); IR: 3131, 3087, 3011, 2949, 1797, 1667, 1616, 1548, 1505 cm^−1^. LCMS (m/z): [M+H, 100%]^+^, 301.1; RT = 2.26 min; HRMS(HESI+) *(m/z)*: [M+Na]^+^ calcd. for C_16_H_10_O_2_N_2_F_2_Na, 323.0603; found, 323.0592, error: 3.3 ppm. ^1^H, ^13^C NMR and IR data corresponds to the literature (4).

**2. Biological supplementary figures**

**A**

**B**

**Figure S1. Squaramide NVR compounds’ effect on inflammasome activation (relates to figure 1)**

LPS-primed (1 μg/mL; 4h) BMDMs were pre-treated with 10 μM NVR compound or vehicle control (DMSO 0.5% v/v) for 15 minutes before addition of 10 μM nigericin. After 90 minutes, culture supernatants were recovered and probed for IL-1β content (**A**) and LDH release (**B**). Data correspond to mean +/- SD of 3 biological repeats. Differences to DMSO control in each group were assessed by Two-way ANOVA and Dunnett’s post-test * p<0.05, ** p<0.01, **** p<0.0001

**A**

**B**

**Figure S2. Dose response curves for selected NVR compounds (relates to figure 2)**

LPS-primed (1 μg/mL; 4h) BMDMs were pre-treated with the stated concentration of NVR compound (0 – 50 μM) or matched vehicle control (DMSO 0 - 2% v/v) for 15 minutes before addition of 10 μM nigericin. After 90 minutes, culture supernatants were recovered and probed for IL-1β content. Data correspond to mean +/- SD of fold change normalised to Nigericin + DMSO condition from 4-5 biological repeats. Differences to a theoretical mean of 1 were assessed by multiple t-tests and corrected for multiple comparisons by Holm-Sidak method * p<0.05, ** p<0.01

**Figure S3. Imiquimod dose response curves for selected NVR compounds (relates to figure 2)**

LPS-primed (1 μg/mL; 4h) BMDMs were pre-treated with the stated concentration of NVR compound (0 – 50 μM) or matched vehicle control (DMSO 0 – 2% v/v) for 15 minutes before addition of 75 μM imiquimod. After 90 minutes, culture supernatants were recovered and probed for IL-1β content. Data correspond to mean +/- SD of fold change normalised to Imiquimod + DMSO condition from 3 biological repeats. Differences to a theoretical mean of 1 were assessed by multiple t-tests and corrected for multiple comparisons by Holm-Sidak method.

NLRP3

Pro-IL1β

β-actin


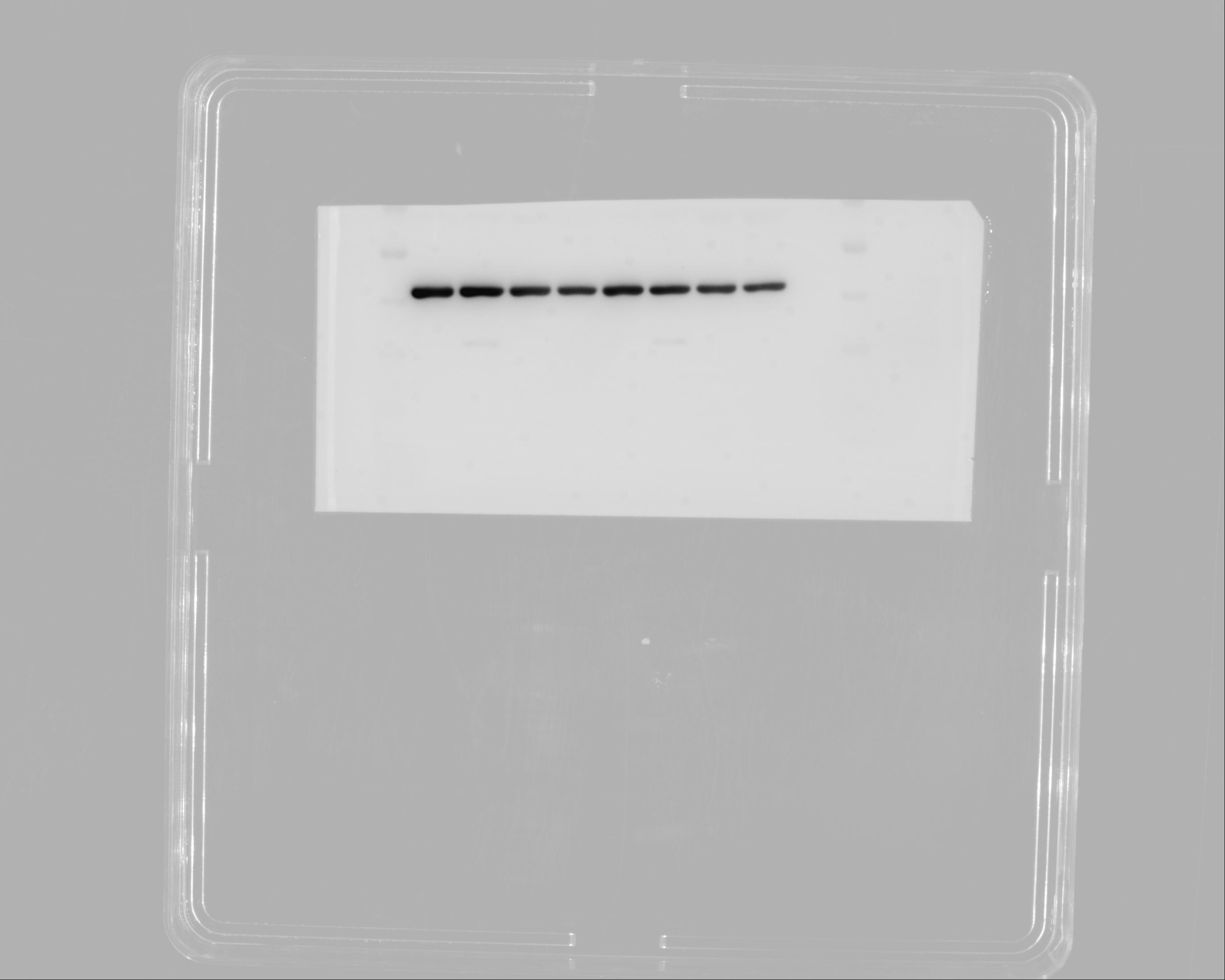

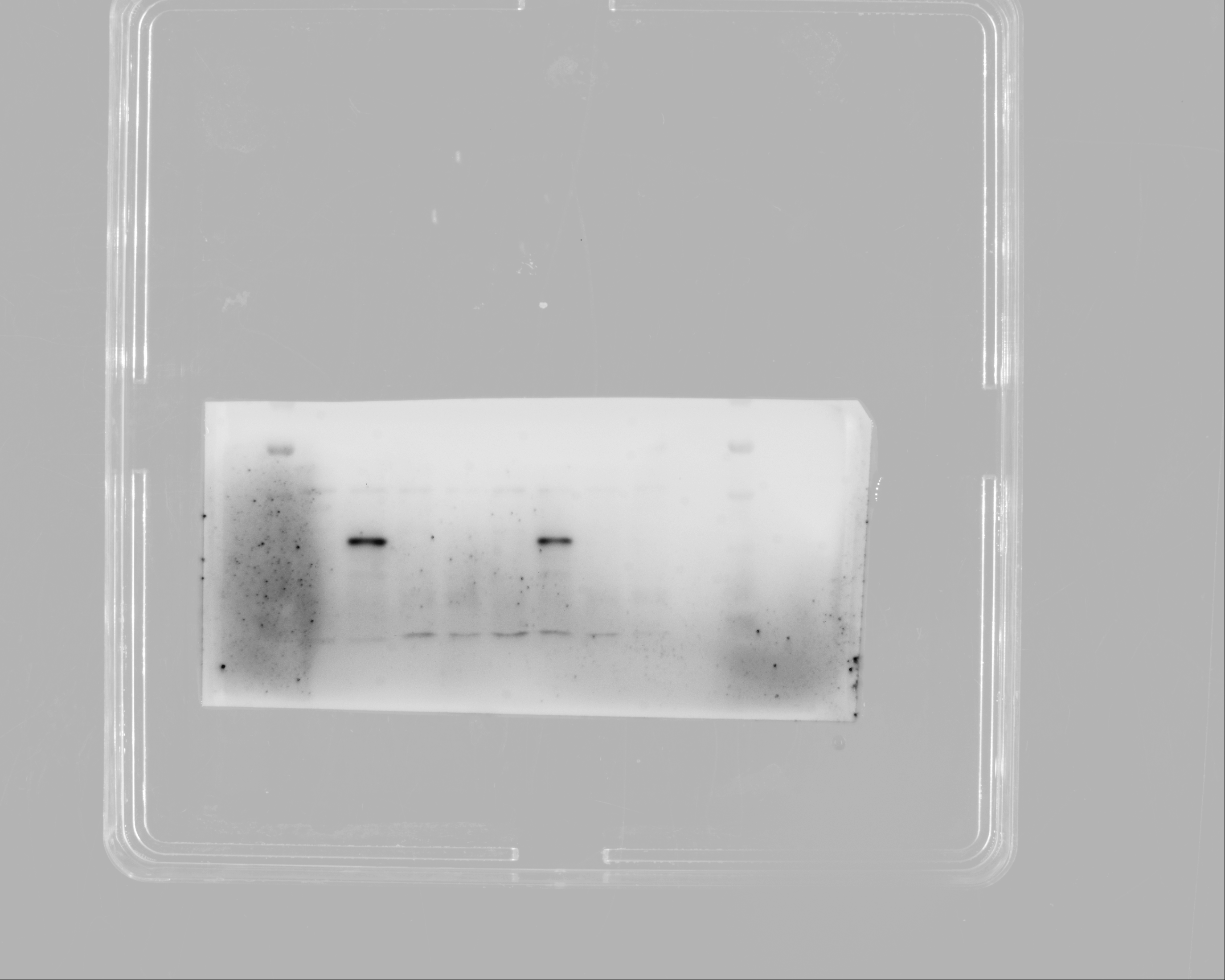

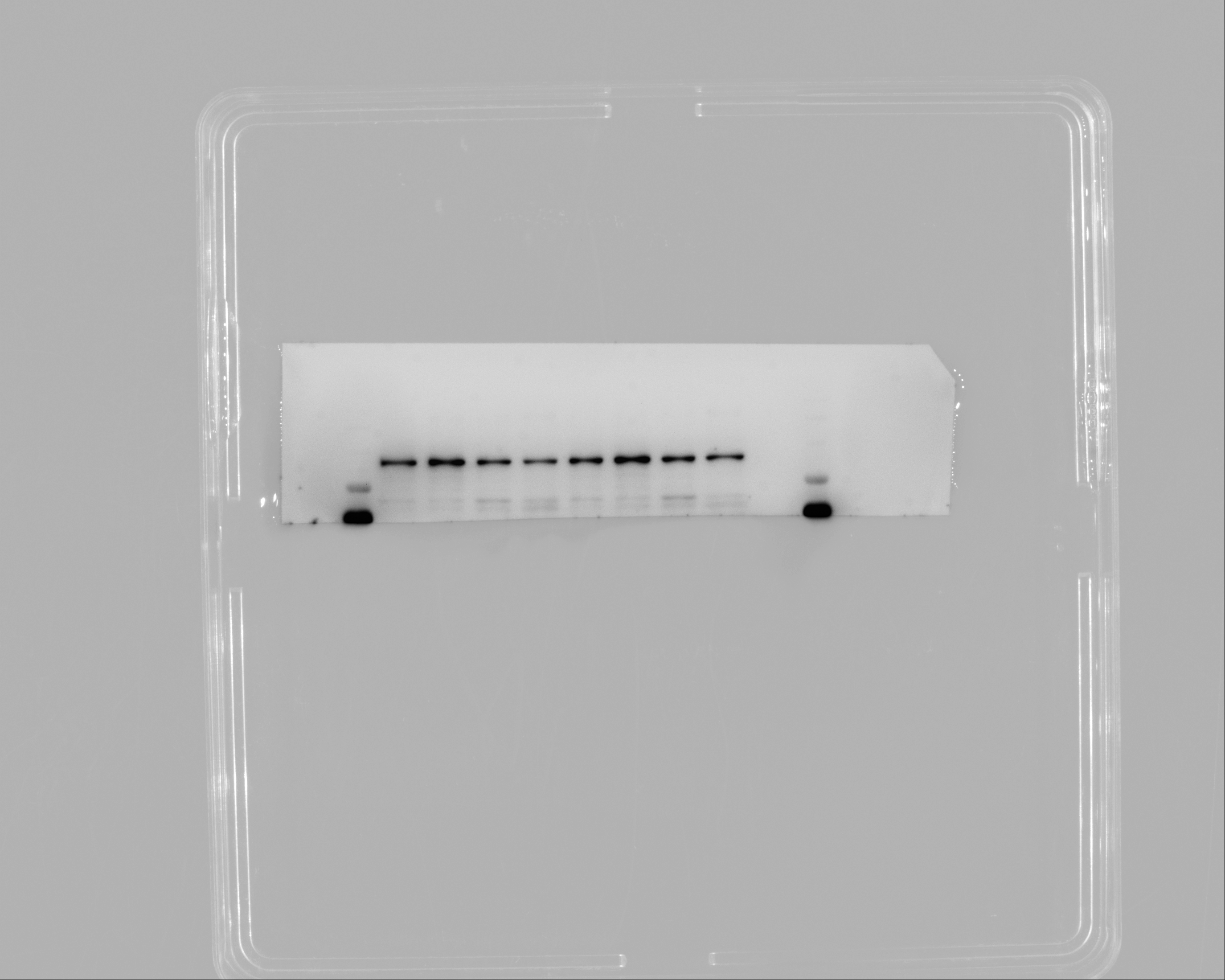


-

LPS

-

LPS

**DMSO**

**Chx**

**Figure S4. Validation of efficacy of cycloheximide as protein synthesis inhibitor (relates to figure 5)**

BMDMs were pre-treated with Cycloheximide (Chx, 10 μg/mL) or vehicle control (DMSO 0.5% v/v) for 30 minutes and subsequently stimulated with LPS (1 μg/mL) for 2 hours. Resulting cell lysates were probed for NLRP3, pro-IL-1β and βactin content by western blot. Data are representative of 3 biological repeats.

**Figure S5. Squaramide compounds do not affect mitochondrial superoxide production (relates to figure 5)**

BMDM were loaded with 5 µM MitoSOX. After 10 minutes, excess dye was washed off and cells were treated with 10 µM Nigericin or vehicle control (EtOH 0.5% v/v) in conjunction with 10 µM NVR77/83 or DMSO control (0.5% v/v). Treatment with 1% H_2_O_2_ was used as a positive control. Mean fluorescence intensity for each condition was recorded 2 hours post-treatment. Data corresponds to mean +/- SD of 3 biological replicates. Differences to NA condition were assessed by Two-way ANOVA and Dunnett’s post-test. ** p<0.01, *** p<0.001, **** p<0.0001.

**3. Computational analysis**

**3.1 Small molecule crystal structure analysis**

The Cambridge crystallographic database was searched for molecules containing the phenyl-substituted substructure shown in Figure S6. This identified 56 structures and the 40 of these that include interactions between squaramides and other species (i.e. not a squaramide-squaramide interaction) were selected for more detailed study.

**Figure S6.** Substructure used for database searching (relates to figure 6)

The structures were inspected manually, and the key interacting groups were identified (Table S1). It is notable that generally crystal structures featuring K^+^ (or Na^+^) include them encased within crown ethers and thus there is little evidence for the ability of squaramide to complex either of these metal ions. In these cases, the CHs of the crown ethers are often found to form interactions with the squaramide carbonyls. When anionic groups such as sulphates, phosphates and nitrates that feature a potential for forming two similar interactions are present, a range of structures in which either one or two interactions are present is observed. DMSO is clearly a privileged group for forming interactions with the NH groups, as are the halide anions F^-^, Cl^-^ or Br^-^.

Notable structures include that with code ZUKDOB in which a bivalent complex with sodium is formed even though it is complexed by a crown ether, HABWEQ and ZUKDUH which see a monovalent interaction with potassium in a crown ether complex. This supports squaramide being able to accommodate Na^+^ comfortably between the two carbonyls in a fashion competitive with crown ether binding whereas K^+^ is able to form an interaction but the bidentate geometry is perhaps less favourable due to the larger size of K^+^ (5).

**Table S1.** Small molecule crystal structures of squaramide complexes.

| **Structure code** | **NH interacting group** | **CO interacting group** | **Comments** |
| --- | --- | --- | --- |
| ASOVIP | O=C (in NMP) | CH_n_NCO (in NMP) |  |
| FAWZAG | Cl^-^ | CH_n_N^+^R_3_ |  |
| FAWZUA | O=S (in DMSO) | CH_3_S=O (in DMSO) |  |
| FAXBUD | Cl^-^ | CH_n_N^+^R_3_ |  |
| FAXCAK | O=S (in DMSO) | CH_3_S=O (in DMSO) |  |
| FAXCEO | Cl^-^ | Alkyl and aromatic CH |  |
| HABWEQ | O=N (x2) | K^+^ (single interaction, K in crown complex) |  |
| HABWIU | Cl-K | K^+^ (single interaction, K in crown complex) |  |
| HABWOA | O=S (x2) | CH_2_O-Na (in crown complex) |  |
| ITUSAU | O=S (in DMSO) | CH_3_S=O (in DMSO) |  |
| ITUSEY | Cl^-^ | CH_n_N^+^R_3_ |  |
| JOTCED | O=S (in SO_4_^2-^) | CH_2_O-Na (in crown complex) |  |
| KAXWUE | O=S (in DMSO) | HCC(CF_3_) in benzene ring |  |
| KAXXAL | O=S (in DMSO) | CH_3_S=O (in DMSO) |  |
| KOJKIF | O=S (in DMSO) | CH_3_S=O (in DMSO) |  |
| LELPUR | Cl^-^ | None |  |
| MEBQIX | O (in water) | HO (in water, single interaction) | Water bridges to adjacent squaramide carbonyl |
| MUYYIP | Br^-^ | CH_n_N^+^R_3_ |  |
| MUYYOV | Cl^-^ | CH_n_N^+^R_3_ (single interaction) |  |
| NIQMUY | Cl^-^ | CH_3_S=O (in DMSO) |  |
| NIQPIP | Cl-Na | CH_2_O-Na (in crown complex) |  |
| NIQPOV | Br-Na | CH_2_O-Na (in crown complex) |  |
| OMUKAJ | O=S (in DMSO) | CH_3_S=O (in DMSO) |  |
| OMUKEN | O=S (in DMSO) | CH_3_S=O (in DMSO) |  |
| QECNOF | None | H_3_CNC=O-Cd |  |
| QELZAM | Cl-K | CH_3_S=O (in DMSO) |  |
| QELZEQ | Br-Na | CH_2_O-Na (in crown complex) |  |
| QUWSEJ | O=CO^-^ (x2) | CH_n_N^+^R_3_ (single interaction) |  |
| QUWSIN | Cl^-^ | CH_n_N^+^R_3_ (single interaction) |  |
| QUWSOT | O (in water) | HO (in water) |  |
| RIHVEM | O=S (in DMSO) | CH_3_S=O (in DMSO) |  |
| RIHVIQ | O=P (x2, in PO_4_^2-^) | CH_n_N^+^R_3_ |  |
| ROXQUS | O=S (in DMSO) | CH_3_S=O (in DMSO) |  |
| UPUQOO | O=C (in DMA) | None |  |
| UPURAB | O (in water) | HO (in water, single interaction) |  |
| UXUTUF | O=C (in DMF) | HC aromatic |  |
| XIDVIS | O (in MeOH) | None |  |
| YAJBIX | F^-^ | CH_2_N^+^R_3_ (x2) |  |
| YAJBOD | F^-^ | HC alkyls |  |
| YAJBUJ | F^-^ | HO (in water, 2 x waters) |  |
| ZUKDOB | Cl^-^ | Na | Na in crown |
| ZUKDUH | Cl^-^ | K | K in crown |

**3.2 Density functional theory calculations**

Calculations were performed at the M06/6-31+G** level incorporating solvation via the IEFPCM with settings appropriate to water in Gaussian16 (6-13). This level of theory has been shown to provide a good balance of accuracy for biomolecular recognition and other properties. Free energies at 1M concentration and 310K were computed using Goodvibes v.3.0.1 (14).

Complexes of individual anions and cations with squaramide were studied first. When anions such as HCO_3_^-^ or phosphates, that could form two interactions with the two NHs, were computed starting from a geometry with two interactions, they optimised to one with only one interaction. The association energy for each species was computed (with no extra corrections for direct interactions between any of these species and the aqueous solvent). These values reveal that there is a distinct preference among the set of anions for binding of chloride. This is consistent with the crystal structure observations and suggests that the squaramide is particularly effective at binding halide anions. The next best anion is phosphate in its dianionic form.

The two cations studied (K^+^ and Na^+^) form complexes that are favoured by 5.24 and 7.51 kcal/mol. This is consistent with the crystal structure evidence suggesting that bivalent binding to sodium occurs even when it is also complexed by crown ether, with potassium also binding in such circumstances but in a monodentate fashion. These binding energies with metals can further polarise the squaramide and enhance the interaction with anions. While chloride remains the favoured anion of the set studied, the two forms of phosphate (mono- and di-anionic) are able to form energetically favoured complexes and this remains the case of mono- and di-alkyl forms of the mono-anion that relate closely to the head groups present in many biological membranes. Acetate, which mimics the fatty acids also present in membranes, also forms a weakly favoured complex in the presence of potassium (or sodium). Sulphate, carbonate and nitrate are not able to form energetically favoured complexes with potassium present.

It is notable that in the two complexes of both potassium and dianionic species (HPO_4_^2-^ and SO_4_^2-^), there is a proton transfer event leading to a complex of a squaramide anion with each of the monoanions (H_2_PO_4_^-^ and HSO_4_^-^).

**Table S2.** Complexation energies between squaramide and a range of anions either alone or in concert with Na^+^, K^+^ or Ca^2+^. Values are free energies in kcal/mol. Values in bold are those computed to be favourable for complexation.

| **Anion** | **Anion only** | **Anion + Na^+^** | **Anion + K^+^** | **Anion + Ca^2+^** |
| --- | --- | --- | --- | --- |
| Cl^-^ | **-2.25** | **-10.25** | **-7.54** | 15.33 |
| HCO_3_^-^ | 2.01 | **-2.49** | 0.41 | 27.54 |
| HPO_4_^2-^ | 1.93 | **-5.15** | **-1.13** | 31.90 |
| H_2_PO_4_^-^ | 3.72 | **-5.03** | **-2.00** | 24.43 |
| MeHPO_4_^-^ | 4.44 | **-4.46** | **-1.53** | 25.08 |
| Me_2_PO_4_^-^ | 4.83 | **-3.60** | **-0.69** | 26.65 |
| NO_3_^-^ | 7.51 | **-0.23** | 2.60 | 29.30 |
| AcO^-^ | 5.14 | **-2.92** | **-0.09** | 24.27 |
| SO_4_^2-^ | 6.06 | 4.41 | 7.38 | 36.83 |
| HSO_4_^-^ | 8.71 | 0.22 | 3.21 | 29.07 |

**Figure S7.** Computational model for NVR77 complexed with potassium (purple) and the different anions from Table S2 (relates to figure 6). Raw data for the coordinates for each atom in the different models are provided as a separate supplementary file.

**4. References**

1. Rostami A, Colin A, Li XY, Chudzinski MG, Lough AJ, Taylor MS. N,N'-diarylsquaramides: general, high-yielding synthesis and applications in colorimetric anion sensing. J Org Chem. 2010;75(12):3983-92.

2. Busschaert N, Kirby IL, Young S, Coles SJ, Horton PN, Light ME, et al. Squaramides as Potent Transmembrane Anion Transporters. Angew Chem Int Edit. 2012;51(18):4426-30.

3. Kumar SP, Glória PMC, Gonçalves LM, Gut J, Rosenthal PJ, Moreira R, et al. Squaric acid: a valuable scaffold for developing antimalarials? MedChemComm. 2012;3(4):489.

4. Pfeifer L, Engle KM, Pidgeon GW, Sparkes HA, Thompson AL, Brown JM, et al. Hydrogen-Bonded Homoleptic Fluoride–Diarylurea Complexes: Structure, Reactivity, and Coordinating Power. J Am Chem Soc. 2016;138(40):13314-25.

5. Zaleskaya M, Karbarz M, Wilczek M, Dobrzycki L, Romanski J. Cooperative Transport and Selective Extraction of Sulfates by a Squaramide-Based Ion Pair Receptor: A Case of Adaptable Selectivity. Inorg Chem. 2020;59(18):13749-59.

6. Frisch MJ, Trucks GW, Schlegel HB, Scuseria GE, Robb MA, Cheeseman JR, et al. Gaussian 16 Rev. C.01. Wallingford, CT2016.

7. Hariharan PC, Pople JA. The influence of polarization functions on molecular orbital hydrogenation energies. Theoretica chimica acta. 1973;28(3):213-22.

8. Hehre WJ, Ditchfield R, Pople JA. Self—Consistent Molecular Orbital Methods. XII. Further Extensions of Gaussian—Type Basis Sets for Use in Molecular Orbital Studies of Organic Molecules. The Journal of Chemical Physics. 1972;56(5):2257-61.

9. Krishnan R, Binkley JS, Seeger R, Pople JA. Self‐consistent molecular orbital methods. XX. A basis set for correlated wave functions. The Journal of Chemical Physics. 1980;72(1):650-4.

10. Tomasi J, Mennucci B, Cammi R. Quantum mechanical continuum solvation models. Chem Rev. 2005;105(8):2999-3093.

11. Zhao Y, Truhlar DG. A new local density functional for main-group thermochemistry, transition metal bonding, thermochemical kinetics, and noncovalent interactions. J Chem Phys. 2006;125(19):194101.

12. Zhao Y, Truhlar DG. Density functionals with broad applicability in chemistry. Acc Chem Res. 2008;41(2):157-67.

13. Zhao Y, Truhlar DG. The M06 suite of density functionals for main group thermochemistry, thermochemical kinetics, noncovalent interactions, excited states, and transition elements: two new functionals and systematic testing of four M06-class functionals and 12 other functionals. Theoretical Chemistry Accounts. 2008;120(1):215-41.

14. Ignacio Funes-Ardoiz RSP. GoodVibes. version 2.0.3 (v2.0.3) ed. Zenodo2018.
