# Supplementary material for: Squaramides enhance NLRP3 inflammasome activation by lowering intracellular potassium
Source: Cell Death Discov. 2023 Dec 22;9:469. doi: 10.1038/s41420-023-01756-9 (PMC10739973; doi:10.1038/s41420-023-01756-9)
Supplement: Supplementary file 2 — Uncropped Western Blots [file 41420_2023_1756_MOESM2_ESM.pptx]

## Slide 1
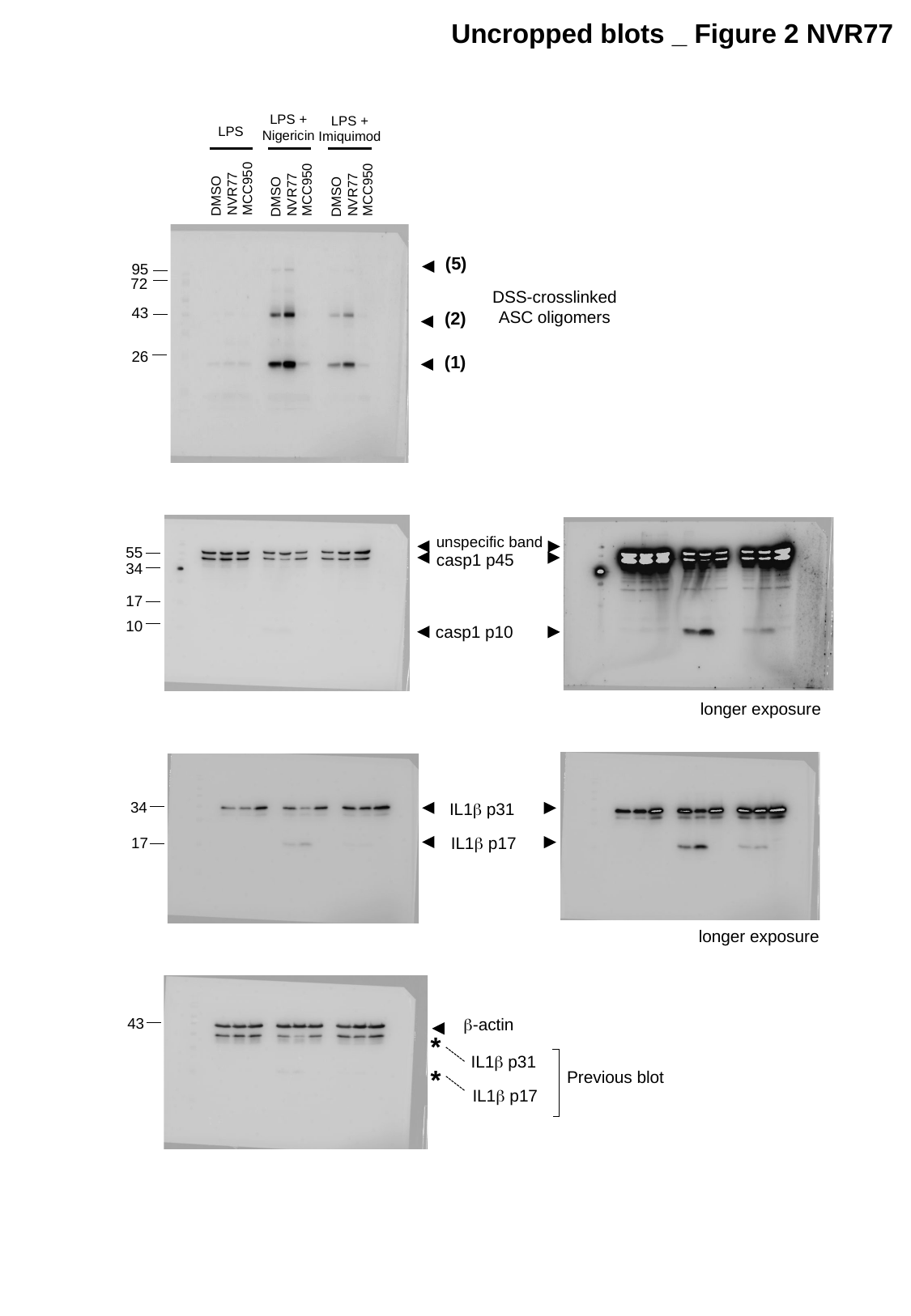

Uncropped blots _ Figure 2 NVR77
LPS +
Nigericin
LPS +
Imiquimod
LPS
MCC950
MCC950
MCC950
NVR77
NVR77
NVR77
DMSO
DMSO
DMSO
(5)
95
72
DSS-crosslinked
ASC oligomers
43
(2)
26
(1)
unspecific band
55
casp1 p45
34
17
10
casp1 p10
longer exposure
34
IL1b p31
IL1b p17
17
longer exposure
43
b-actin
*
IL1b p31
*
Previous blot
IL1b p17

## Slide 2
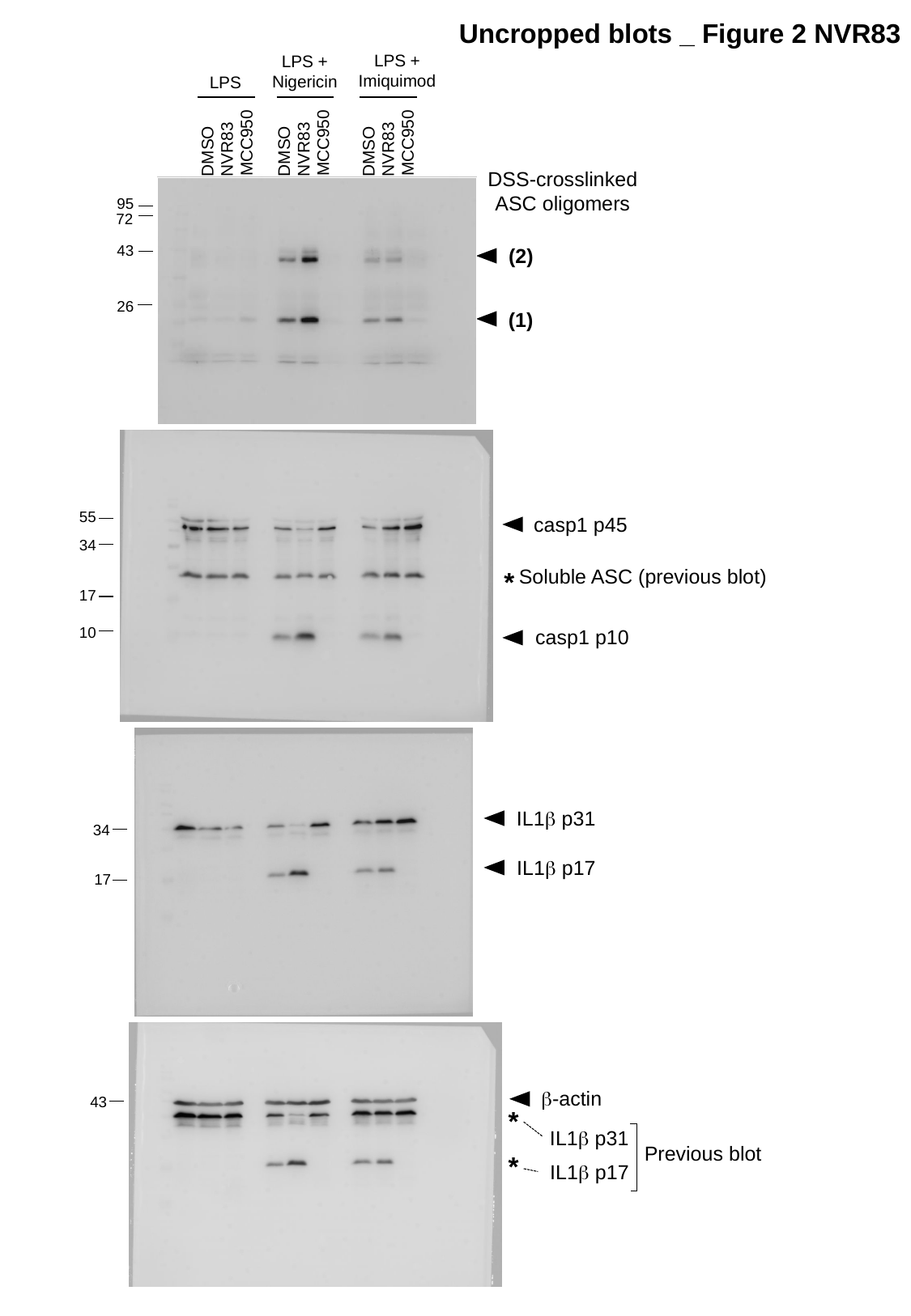

Uncropped blots _ Figure 2 NVR83
LPS +
Imiquimod
LPS +
Nigericin
LPS
MCC950
MCC950
MCC950
NVR83
NVR83
NVR83
DMSO
DMSO
DMSO
DSS-crosslinked
ASC oligomers
95
72
43
(2)
26
(1)
55
casp1 p45
34
Soluble ASC (previous blot)
*
17
10
casp1 p10
IL1b p31
34
IL1b p17
17
b-actin
43
*
IL1b p31
Previous blot
*
IL1b p17

## Slide 3
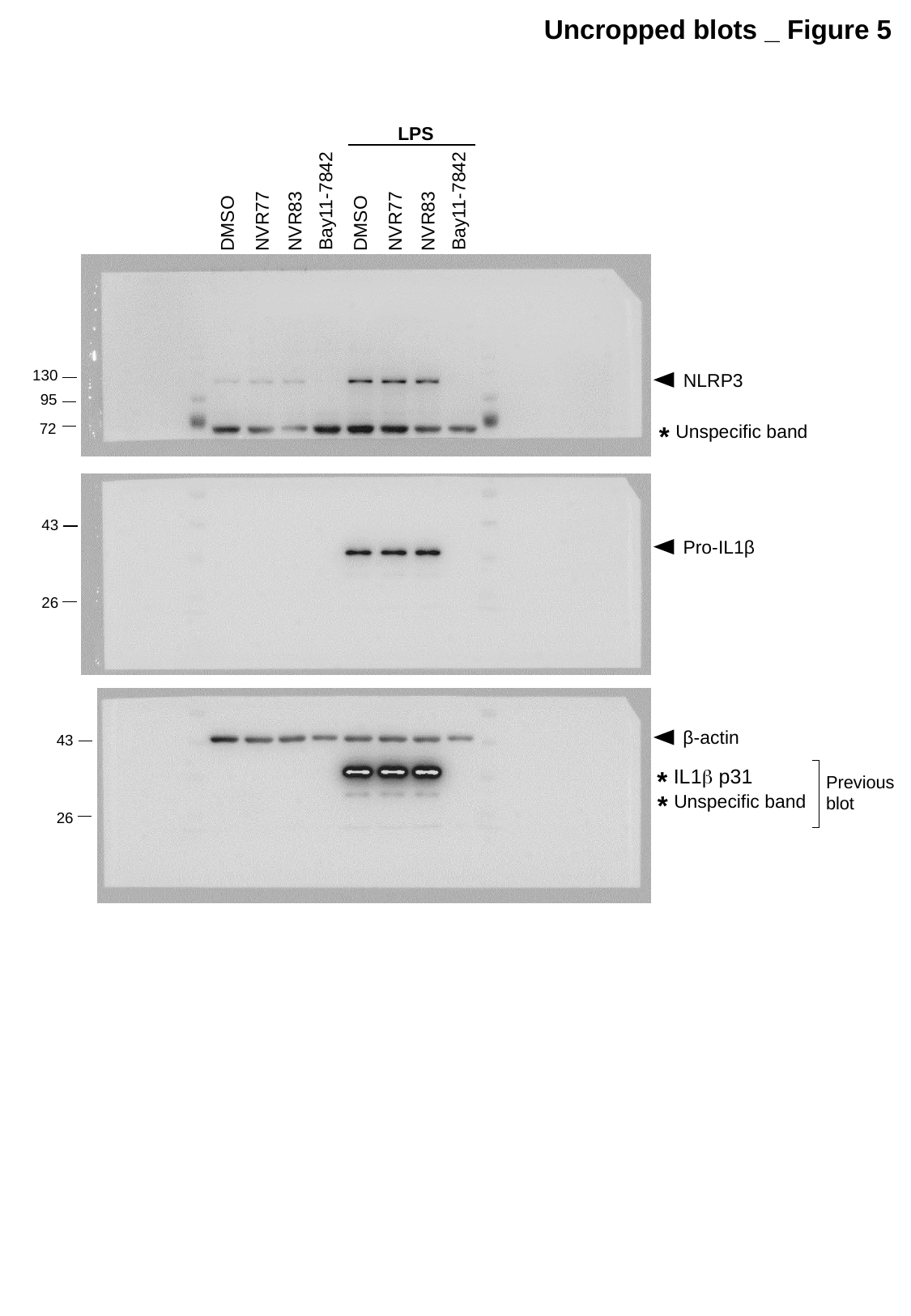

Uncropped blots _ Figure 5
LPS
Bay11-7842
Bay11-7842
NVR77
NVR83
NVR77
NVR83
DMSO
DMSO
130
NLRP3
95
*
72
Unspecific band
43
Pro-IL1β
26
β-actin
43
IL1b p31
*
Previous blot
*
Unspecific band
26

## Slide 4
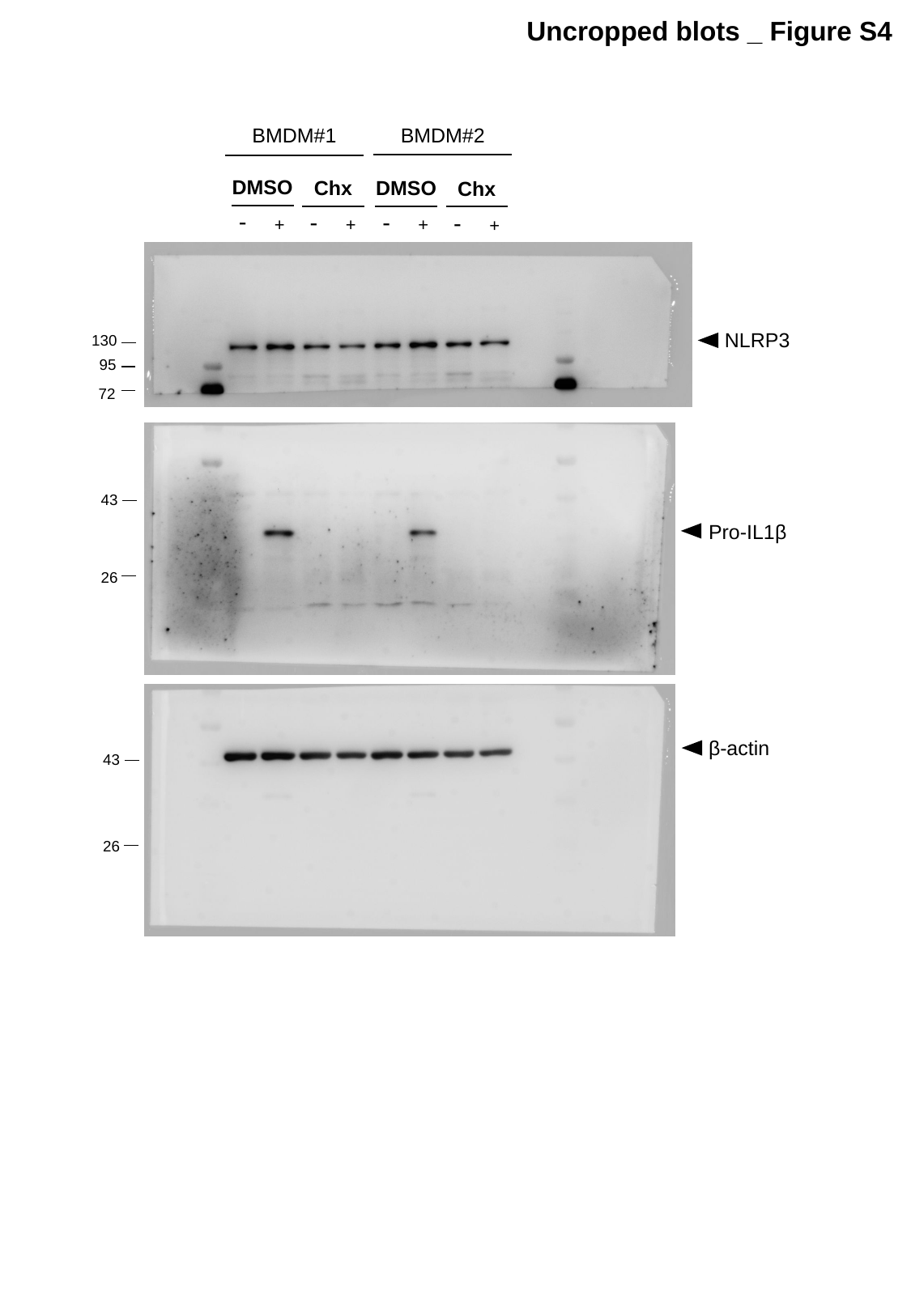

Uncropped blots _ Figure S4
BMDM#2
BMDM#1
DMSO
Chx
DMSO
Chx
-
-
-
-
+
+
+
+
NLRP3
130
95
72
43
Pro-IL1β
26
β-actin
43
26
